# A predicted model-aided reconstruction algorithm for X-ray free-electron laser single-particle imaging

**DOI:** 10.1107/S2052252524004858

**Published:** 2024-06-21

**Authors:** Zhichao Jiao, Yao He, Xingke Fu, Xin Zhang, Zhi Geng, Wei Ding

**Affiliations:** a Laboratory of Soft Matter Physics Institute of Physics, Chinese Academy of Sciences Beijing100190 People’s Republic of China; b Beijing Synchrotron Radiation Facility Institute of High Energy Physics, Chinese Academy of Sciences Beijing100049 People’s Republic of China; c University of Chinese Academy of Sciences Beijing100049 People’s Republic of China; d Research Instrument Scientist New York University Abu Dhabi Abu Dhabi United Arab Emirates; e The University of Hong Kong Hong Kong SAR People’s Republic of China; Harima Institute, Japan

**Keywords:** single-particle imaging, X-ray free-electron lasers, 3D reconstruction, phase problem, protein structures, single particles, molecular orientation determination, XFELs

## Abstract

Ultra-intense, ultra-fast X-ray free-electron lasers (XFELs) enable the imaging of single protein molecules under ambient temperature and pressure. A crucial aspect of structure reconstruction involves determining the relative orientations of each diffraction pattern and recovering the missing phase information. In this paper, we introduce a predicted model-aided algorithm for orientation determination and phase retrieval, which has been tested on various simulated datasets and has shown significant improvements in the success rate, accuracy and efficiency of XFEL data reconstruction.

## Abstract 2

A predicted model-aided reconstruction algorithm is proposed for orientation determination and phase retrieval in X-ray free-electron laser single-particle imaging. The predicted model-aided algorithm exhibits significant improvements in the success rate, accuracy and efficiency of the reconstruction process.

## 1. Introduction

Real knowledge about 3D atomic localizations inside biological molecules revealed by X-ray crystallography has dramatically advanced our understanding of life sciences across the world (Garman, 2014 ▸). Despite such unprecedented resolving power of X-ray crystallography, the requirement for stringent periodic arrangement of protein molecules poses a fundamental challenge for large proteins that are difficult to crystallize. Recent advances in cryo-electron microscopy (cryo-EM) allow for high-resolution structural determination of sufficiently large proteins or macromolecular assemblies by directly imaging individual protein particles that are embedded in vitreous ice (Cheng *et al.*, 2015 ▸), thus circumventing the need for crystallization. However, the cryo-preserved protein particles commonly used for cryo-EM may undergo some conformational changes that are likely to deviate from its native state at room temperature resulting from rapid freezing (Bock & Grubmüller, 2022 ▸) and radiation-induced damage (Baker & Rubinstein, 2010 ▸; Glaeser, 2016 ▸). In contrast, single-particle X-ray diffraction equipped with extremely intense and ultrashort X-ray free-electron lasers (XFELs) aims to open up a novel avenue in structural biology by capturing snapshot diffraction signals of an individual protein in its native solution state while miantaining physiological temperature before radiation damage takes place and destroys the particle (Neutze *et al.*, 2000 ▸; Chapman *et al.*, 2006*a* ▸; Seibert *et al.*, 2011 ▸).

Theoretical studies have suggested that near-atomic 3D resolution of isolated non-crystalline protein particles should be possible with current XFEL sources when millions of diffraction patterns are available (Miao *et al.*, 2001 ▸; Bortel *et al.*, 2009 ▸; Gaffney & Chapman, 2007 ▸). In practice, most XFEL experiments performed on individual macromolecules to date have been limited to a 3D resolution generally below the nanometre scale, albeit using giant viruses. The first 3D reconstruction of biological particles from experimentally measured XFEL diffraction patterns is achieved with a full-period resolution of 125 nm using the Giant Mimivirus, one of the largest viruses spanning approximately 450 nm in diameter (Ekeberg *et al.*, 2015 ▸). From then on, more and more successful 3D reconstructions of smaller viruses have been reported at much better resolutions. For example, the XFEL single-particle experiment has provided 3D structural information about the Melbourne virus whose diameter is around 230 nm at 28 nm resolution (Lundholm *et al.*, 2018 ▸). Moreover, even smaller viruses, such as Rice Dwarf Virus (Kurta *et al.*, 2017 ▸; Munke *et al.*, 2016 ▸) and bacteriophage PR772 (Assalauova *et al.*, 2020 ▸; Reddy *et al.*, 2017 ▸), both with a diameter of around 70 nm, have also been structurally determined to resolutions of 17 nm and 6.9 nm, respectively. These proof-of-principle XFEL experiments also highlight the fact that the weak diffraction signals of single particles, limited number of high-quality diffraction patterns coupled with sample heterogeneity will eventually limit the achievable resolution.

In single-particle diffractive imaging (SPI), ensembles of identical particles are successively delivered into the XFEL beam at random orientations through either a liquid medium (DePonte *et al.*, 2008 ▸) or aerosolization (Hantke *et al.*, 2014 ▸). To build up a continuous 3D diffraction intensity in reciprocal space, the orientation of each particle must be recovered *a posteriori*. Hitherto, there have been three major strategies to computationally estimate the orientations of XFEL diffraction patterns. Earlier methods mainly focus on finding the common lines or arcs among diffraction patterns which can provide relative orientation information when the signal-to-noise ratio (SNR) is sufficiently high (Shneerson *et al.*, 2008 ▸; Bortel & Tegze, 2011 ▸; Yefanov & Vartanyants, 2013 ▸). A more sophisticated method is based on the manifold-embedding technique (Fung *et al.*, 2008 ▸) which holds the promise of mapping the continuous conformational landscape in a biological system (Hosseinizadeh *et al.*, 2017 ▸). Another of the most widely used methods is built on the framework of projection matching (van Heel, 1984 ▸; Penczek *et al.*, 1994 ▸) by iteratively alternating between orienting 2D diffraction patterns and updating 3D diffraction intensity, such as the correlation maximization (CM) algorithm (Tegze & Bortel, 2012 ▸, 2021 ▸) and the expansion maximization compression (EMC) algorithm (Loh & Elser, 2009 ▸; Ayyer *et al.*, 2016 ▸). However, the accuracy of orientation determination based on projection matching is heavily dependent on the number of diffraction patterns (Poudyal *et al.*, 2020 ▸) together with the incident pulse fluences (Ayyer *et al.*, 2019 ▸; E *et al.*, 2022 ▸); therefore, the current methods are prone to failure under a number of difficult circumstances including the aforementioned limited data intensity and weak diffraction signals.

After assembling 2D diffraction patterns into a 3D intensity with recovered orientations, the next important step is to reconstruct electron-density distribution from only intensity information via phase retrieval methods. Under the oversampling conditions, the 3D intensity can be computationally phased through iteratively enforcing constraints in both real space and reciprocal space (Miao *et al.*, 1999 ▸; Marchesini *et al.*, 2003 ▸; Chapman *et al.*, 2006*b* ▸). Despite its effectiveness, the success and quality of reconstruction are highly susceptible to the central data within 3D diffraction intensity (Nishino *et al.*, 2003 ▸; Miao *et al.*, 2005 ▸). This is because the approximate shape of the reconstructed structure is mathematically defined by low-resolution diffraction data which have been extensively demonstrated to be crucial for successful *ab initio* phase recovery (Subbiah, 1991 ▸; Lunin *et al.*, 2000 ▸; Jorda *et al.*, 2016 ▸; Jiang *et al.*, 2023 ▸). Unfortunately, the loss of central data is essentially unavoidable since the gaps between detector rows need to be large enough to directly pass the incident XFEL beam. Furthermore, as the resolution approaches near-atomic level, a particular problem may arise that even a small amount of central data can account for a relatively large proportion at low resolution, the absence of which can make direct phase retrieval difficult and sometimes impossible.

As stated previously, current orientation determination and phase retrieval procedures generally employ iterative algorithms that are initiated from a random solution and inevitably require a substantial number of iterations to reach convergence. In some more challenging cases, both procedures may even get stuck at a local minimum and fail to find the correct solution, thus necessitating the use of some prior structural information about the particles. This idea is analogous to the single-particle analysis in cryo-EM where a good estimate of the initial model generated from *ab initio* 3D reconstruction or homologous structure can obviously facilitate orientation determination (Harauz & Ottensmeyer, 1984 ▸, 1983 ▸), supervised classification (Gao *et al.*, 2004 ▸; Heymann *et al.*, 2004 ▸; Brink *et al.*, 2004 ▸) and reference-based refinement (Grigorieff, 2007 ▸; Scheres, 2012 ▸; Punjani *et al.*, 2017 ▸). Recent technological breakthroughs in machine-learning based *AlphaFold2* are pushing the prediction accuracy of protein structures to an unparalleled level that is on par with experimental structural quality (Jumper *et al.*, 2021 ▸; Tunyasuvunakool *et al.*, 2021 ▸). The highly real predicted models have found widespread applications in a variety of protein structure determination techniques, such as cryo-EM (Hryc & Baker, 2022 ▸; Mosalaganti *et al.*, 2022 ▸; Skalidis *et al.*, 2022 ▸), X-ray crystallography (Hu *et al.*, 2022 ▸; Zhao *et al.*, 2023 ▸; Millán *et al.*, 2021 ▸) and nuclear magnetic resonance (NMR) (Fowler & Williamson, 2022 ▸; Tejero *et al.*, 2022 ▸). However, to our knowledge, the application of high-quality predicted models to SPI reconstruction remains unexplored.

In this study, we further incorporate the prior knowledge of predicted models into SPI to enhance and speed up 3D reconstructions of single particles. In our method, a predicted model is converted to the regularly sampled reciprocal intensity in accordance with the diffraction geometry relationship. The reciprocal-space intensity serves as a starting reference to initiate the iterative orientation determination and phase retrieval procedures with its intensity and phase information, respectively. Our method is numerically demonstrated to be more tolerant to limited data intensity, weak diffraction signals and unmeasured low-resolution data.

At the time of writing, experimental determination of 3D macromolecular structures with XFELs is restricted to protein nanocrystals and giant virus particles. Nevertheless, ongoing efforts to observe single protein molecules are underway (Ekeberg *et al.*, 2022 ▸), driven by the continuous increase in brightness and repetition rate of European XFEL or LCLS-II sources (Decking *et al.*, 2020 ▸; Sobolev *et al.*, 2020 ▸), as well as advancements in detector technology and efficient sample delivery methods. It is anticipated that our proposed method will enable experimental achievement of high-resolution 3D reconstructions from individual protein particles of moderate sizes.

## 2. Methods

### 2.1. Orientation determination

**Figure 1:**
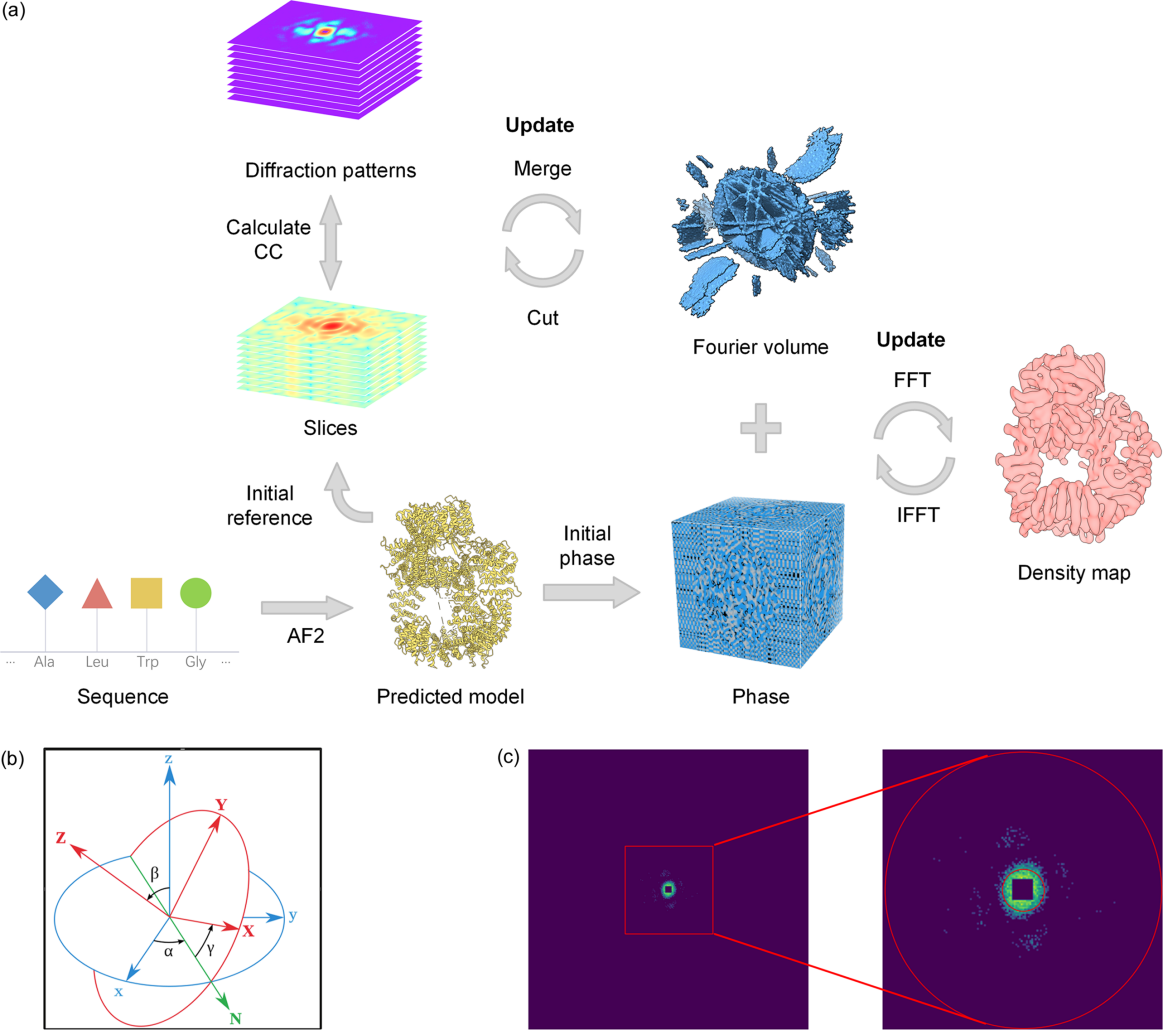
(*a*) Flowchart of the orientation determination algorithm as well as the 3D phase recovery algorithm. The orientation determination algorithm begins with a roughly accurate predicted model, which provides an initial reference Fourier volume. The volume will be iteratively improved with updated orientation information. The above procedure will be performed multiple times until convergence is reached. The predicted model also provides an initial set of phases instead of random ones for the conventional phase retrieval algorithm, which iterates back and forth in dual-space to recover the final electron-density map. (*b*) Definition of rotation, with three Euler angles α, β and γ under a kinematic coordinate system. More specifically, the first angle α indicates rotation around the blue *z* axis, which also brings the blue *x* axis to the green *N* axis. The second angle β indicates rotation around the green *N* axis, which further brings the blue *z* axis to the red *Z* axis. The third angle γ indicates the rotation around the red *Z* axis. If we imagine that the circle represents a 2D Fourier slice before and after 3D rotation, then the *Z* axis becomes its normal axis. Of note, the incident X-ray is in the red *Z* axis direction. (*c*) Example of the simulated diffraction pattern in this study, and the upper and lower limits of the resolution circles for calculating Pearson correlation are also shown.

The classical CM algorithm (Tegze & Bortel, 2012 ▸) was employed for orientation determination of each diffraction pattern in this study. Traditionally, the CM algorithm is initiated with a random 3D intensity distribution, necessitating many more iterations to reach convergence and sometimes even leading to failure. Taking advantage of the predicted structures, it is possible to push the initial trial solution close to the global minimum and consequently circumvent the above challenges. The proposed orientation determination workflow is illustrated in Fig. 1 ▸(*a*) and the detailed steps are elaborated as follows.

(i) Preprocessing the diffraction patterns. The diffraction pattern must be preprocessed before determining its orientation. First, in order to enhance the SNR and further improve computational efficiency, each diffraction pattern is binned by a factor of 2. Of note, the binning operation means taking 2 × 2 adjacent pixels and summing of their values into a new value. Second, the binned diffraction pattern should be projected onto the Ewald sphere in reciprocal space (abbreviated to experimental Fourier slice) according to the diffraction geometry relationship. Third, the experimental Fourier slice is embedded into a 3D Fourier volume whose sampling interval and dimension are determined by the maximum scattering vector, detector size and binning factor. Since the 3D coordinates of the Fourier slice can hardly coincide with the uniform 3D grid of the reference volume, 3D linear interpolations are applied for such coordinate transformation.

(ii) Generation of 3D reference Fourier volume from the predicted model. First, the predicted model is centered and transformed into an electron-density map by broadening and superposing each model atom at a spacing of 1 Å. Note that the atomic electron distribution is subject to a 3D Gaussian function with a default standard deviation of 1 Å, similar to the strategy adopted by the *Sfall* program within the *CCP4* software suite. Subsequently, the map is low-pass filtered and converted to a regularly sampled 3D Fourier volume whose sampling interval and dimensions must be consistent with those of the experimental Fourier slice in reciprocal space. The calculated 3D Fourier volume with both amplitude and phase information then serves as the reference for guiding both orientation determination and phase recovery procedures.

(iii) Finding the best orientation for each diffraction pattern. Given the reference Fourier volume, the orientation of each diffraction pattern can be determined by seeking its maximum correlation with all possible Fourier slices extracted from the reference Fourier volume. To this end, how to sample the whole orientation space becomes of critical significance for balancing the contradiction between orientation accuracy and computational efficiency. In our algorithm, the orientation of each possible Fourier slice is defined by three Euler angles: α, β and γ, each of which rotates the object under the kinematic coordinate system [Fig. 1 ▸(*b*)]. In order to span the whole orientation space, the scope of each Euler angle should be defined to 2π, π, 2π, respectively. Additionally, to ensure a uniform sampling across the 3D orientation space, the angular interval for α and β must satisfy the following relationship (Powell, 1999 ▸):
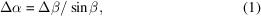
where Δα and Δβ indicate the equidistant angular step of α and β, respectively. In this study, Δβ is set to 0.1 radians by default. As shown in Fig. 1 ▸(*b*), the first two Euler angles α and β will align the normal axis of the Fourier slice with the incident X-ray direction, whereas the third Euler angle γ in essence solely accounts for a self-rotation around the incoming beam. However, if the object is rotated about the axis of the primary beam, the diffraction pattern will not alter except for an in-plane rotation. Therefore, the angle γ will be treated differently from the other two angles. And the 3D orientation space can be divided into the 2D subspace, defining the normal axis of the Fourier slice and the 1D subspace encoding the self-rotation around this normal axis. As a result, it is feasible to only exhaustively search in the direction of the normal axis of the Fourier slice (angles α and β) and the in-plane rotation (angle γ) around the normal axis can be quickly determined based on the cross-correlation theorem.

After assigning the rotational operations subtending the whole orientation space, all possible Fourier slices excluding the self-rotated ones are subsequently extracted from the reference volume with the same sampling interval and dimension as the experimental Fourier slice. The similarity of any extracted Fourier slice with a specified experimental diffraction pattern can be mathematically expressed in the form of Pearson correlation. Obviously, the orientation with the maximum similarity score will be assigned to the diffraction pattern. To make use of the cross-correlation theorem for calculating the Pearson correlation between Fourier slices and the diffraction pattern, both defined on a Cartesian grid should be converted to a series of concentric circles defined on a polar grid, expressed as *I*_R_(*r*, θ). Here the subscript R represents the rotation of the normal axis defined by two Euler angles α and β, the parameter *r* denotes the radial radius and the parameter θ indicates the azimuthal angle. To further make the computation as effective as possible, each Fourier slice *I*_R_(*r*, θ) is normalized independently for each resolution circle given by *r_i_* to avoid domination of the high-intensity, low-resolution region. The normalization is performed according to the following criterion:
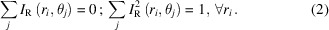
Given a self-rotation angle γ, the Pearson correlation of a specific diffraction pattern with an extracted Fourier slice can be expressed as
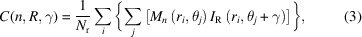
where *M*_*n*_ signifies the *n*th normalized diffraction pattern defined on a polar grid, *I*_*R*_ denotes an extracted Fourier slice from the reference volume by a combined rotation of *R* and γ, and *N*_r_ indicates the number of resolution circles which are summed to yield the Pearson correlation. Generally, the very low-resolution data are intense enough to easily overweigh the Pearson correlation while the very high-resolution data exhibit a sufficiently poor SNR, both of which could deteriorate the accuracy of orientation. As a result, both regions should be excluded from the outer summation in equation (3). The upper and lower limits of resolution circles are depicted in Fig. 1 ▸(*c*), which can be dynamically adjusted based on the specified pattern. To increase efficiency, the fast Fourier transform (FFT) algorithm is applied to calculate equation (3) for all self-rotation angles (γ) at once according to the cross-correlation theorem as follows:
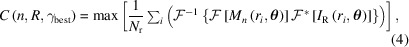
where γ_best_ defines the self-rotation angle γ where the Pearson correlation is at a maximum given the rotation *R*, **θ** is a vector of azimuthal angles with a range of 2π, the operator 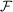 means 1D FFT about vector **θ**, 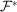 means the conjugate of 1D FFT and 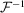 is the inverse 1D FFT.

For each possible rotation *R*, the maximum Pearson correlation is calculated through equation (4). And the best Euler angles for a specified diffraction pattern are obtained as follows:
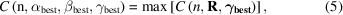
where α_best_, β_best_, γ_best_ define the Euler angles where the Pearson correlation is at a maximum; **R** is a vector of all possible rotations about the normal axis of the Fourier slice; and **γ_best_** is a vector of best self-rotation angle corresponding to each element of **R**.

(iv) Merging each diffraction pattern into an updated Fourier volume with the Euler angles assigned as described above. On finding the best Euler angles for all diffraction patterns, an updated Fourier volume will be generated by accumulating every diffraction pattern into the same 3D volume according to the best Euler angles.

(v) Iterate between step (iii) and step (iv) until convergence is achieved or a predefined number of iterations is reached. Thereafter, the converged Fourier volume will be refined by decreasing the sampling interval Δβ of the Euler angle to 0.02 radians.

To accelerate the above calculation, MPI based parallelism is performed by dividing the whole orientation space into different parts and allocating different jobs of orientation determination to multiple computer cores.

### 2.2. 3D phase recovery

Prior to phasing the oversampled Fourier volume, the 3D amplitudes must be derived by directly taking the square root of the intensity volume. In order to compensate for the missing low-resolution data, a cubic box roughly encapsulating the protein molecule is deduced based on the size of the target protein and diffraction resolution. Afterwards, a hybrid HIO and ER (Fienup, 1982 ▸) algorithm coupled with the above-defined box as real-space support is employed for recovering phase information pertaining to the 3D amplitudes. For comparison, the phase retrieval algorithm is initiated with random phases and calculated phases from the predicted model. Note that the calculated 3D phases must be mapped accurately onto the Fourier volume at a voxel level so that an accurate initial electron-density map is guaranteed. For a monomeric structure, this is not a problem since the orientation of the Fourier volume is uniquely determined by the reference predicted model and the phases are directly calculated from the same predicted model. However, in cases where we only use part of the predicted model to generate the reference volume for a multimeric or multi-domain structure, the final converged Fourier volume can deviate significantly from the reference volume and the calculated phases from predicted model may no longer match the Fourier volume.

The detailed HIO-ER algorithm is described as follows. In combination of the initial phase set with the 3D amplitudes, a reciprocal-space complex array is obtained. By applying inverse FFT to the complex array, a real-space complex array is created. The real part of the real-space array corresponds to the electron-density map and the imaginary part is directly set to zero. The electron density outside the above-defined support and the negative electron density inside the support are slowly pushed to zero. By applying FFT to the modified real-space array, an updated reciprocal-space array is generated. The modulus of the updated reciprocal-space array is replaced with the 3D amplitudes, whereas the phases and the missing low-resolution data are kept unchanged. This process creates a new reciprocal-space array, which is used for the next iteration. On convergence, the electron-density map within the support is extracted and converted to a reciprocal-space array by FFT, which is further padded with zeros. As a result, a much more finely sampled electron-density map with a spacing of 1 Å can be obtained by inverse FFT of the zero-padded array. Eventually, the real structure is docked into the electron-density map by rigid-body fitting in *Chimera* (Pettersen *et al.*, 2004 ▸).

### 2.3. Simulation of diffraction patterns

We tested the above algorithms using simulated diffraction patterns of several biomolecules under different conditions. In theory, each diffraction pattern corresponds to a central spherical cap resulting from the Ewald sphere that intersects the intensity volume in reciprocal space. Specifically, the position of each pixel on the detector plane can be represented by a scattering vector **q** in reciprocal space and its intensity was calculated pixel by pixel based on the following formula
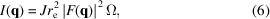
where *J* is the incident X-ray photon fluence, *r*_e_ is the classical electron radius, Ω is the solid angle subtended by the corresponding pixel on the detector and *F*(**q**) denotes the structure factor of the protein molecule. The structure factor can be calculated by performing a Fourier transform of the electron-density distribution inside the molecule according to far-field diffraction theory. Of note, in order to avoid interpolation errors induced by the transformation from a regular 3D Cartesian grid to a curved Ewald sphere, non-uniform fast Fourier transform (NUFFT) (Geng *et al.*, 2021 ▸; Fessler & Sutton, 2003 ▸) was especially adopted to yield accurate structure factors from electron-density maps without markedly compromising computational efficiency. The orientation of each diffraction pattern was determined by rotating the Ewald sphere in reciprocal space according to three randomly generated Euler angles where the third angle indicates in-plane rotation along the incident X-ray direction. In order to mimic real-world situations, Poisson noise was further added to each diffraction pattern and a number of intensities at the center of the diffraction patterns were also removed with different sizes to simulate the effect of beam stop that blocks the direct X-ray beam.

For simulation purposes, there are several critical parameters that need to be carefully examined in order to comply with the experimental setup as closely as possible, such as XFEL wavelength, incident pulse fluences, sample-to-detector distance and detector size. As a result, the specific simulation parameters used in this study are listed in Table 1 ▸. Note that in order to evaluate the influence of varying pulse fluences as well as the number of diffraction patterns on our reconstruction algorithm, different combinations of simulation parameters are used for comparison.

### 2.4. Test data

**Figure 2:**
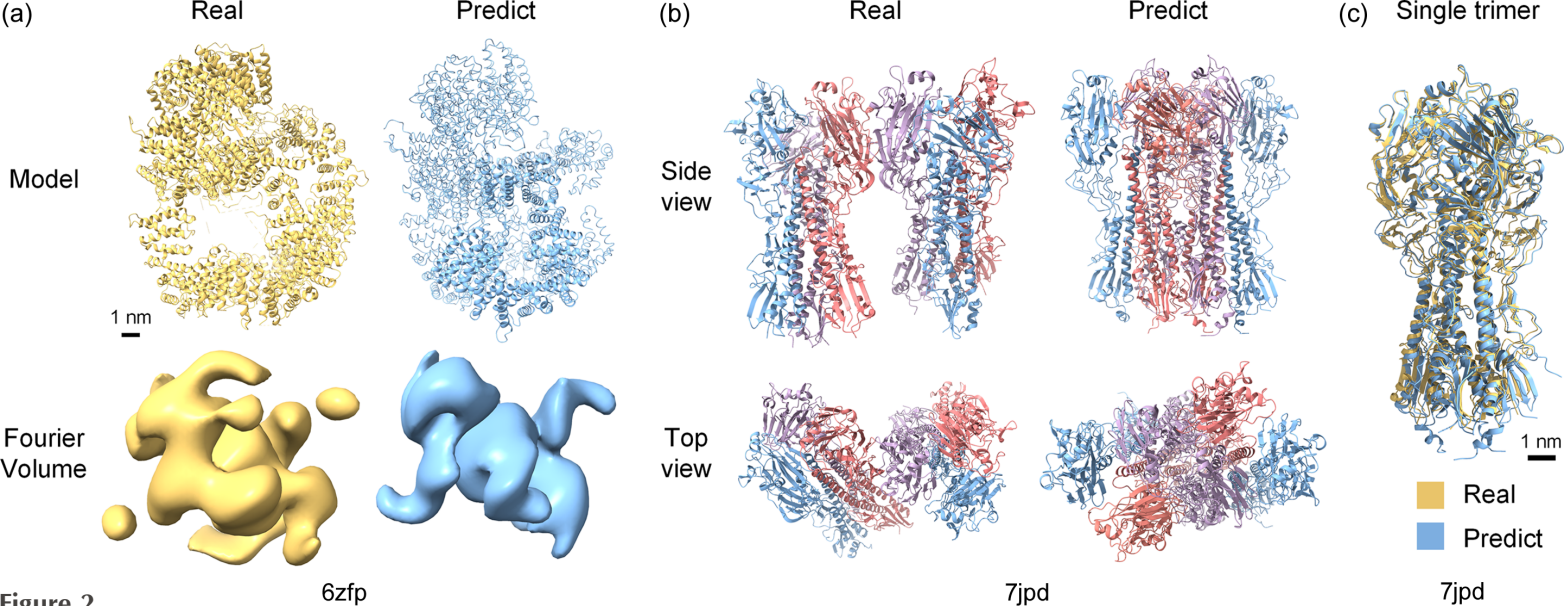
Structural comparison between the predicted model and the real protein model. (*a*) Real and predicted models of test protein 6zfp. Their corresponding Fourier volumes are also displayed for comparison. (*b*) Real and predicted models of test protein 7jpd viewed from two directions. The top row shows the side view of the models, and the bottom row shows the top view of the models. (*c*) Real and predicted single trimer models of test protein 7jpd. The yellow one represents the real model, and the blue one represents the predicted model.

In order to simulate a single-molecule diffraction experiment, two protein structures with the PDB entries 6zfp (Chaplin *et al.*, 2021 ▸) and 7jpd (Ghafoori *et al.*, 2023 ▸) were selected for testing. Test protein 6zfp is a very large monomer, comprising a total of 3704 residues with a molecular weight of 472.06 kDa; it is a DNA-dependent protein kinase catalytic subunit (DNA-PKcs), which is a key protein involved in the DNA repair process. The corresponding predicted model was obtained directly from the *AlphaFold2* database (https://alphafold.com/). Note that the complete sequence was divided into 15 short peptide chains of lengths of 1400, with a repetitive sequence of length 1000 between adjacent peptide chains. Therefore, we docked all the peptide chains into a single complete model based on the repetitive segment. The final model is illustrated in Fig. 2 ▸(*a*). A visual comparison reveals that the predicted model shares a rough structural similarity with the actual model. However, notable discrepancies appear in terms of the specific structural details, thus resulting in a considerably high root mean square deviation of 9.425 Å.

*AlphaFold2* generally performs worse when predicting protein complexes consisting of many chains. To this end, the protein 7jpd, which is made up of 12 chains in the form of two identical trimers, was further chosen as a test case. Protein 7jpd is a full-length mature hemagglutinin from influenza A virus. Hemagglutinin is a surface glycoprotein of the influenza virus that mediates the entry of the virus into host cells by binding to sialic acid receptors and is a primary target for antibody responses and vaccine development. The predicted model of 7jpd was predicted on a local workstation with the input sequence of all 12 chains. As shown in Fig. 2 ▸(*b*), the predicted complex model deviated significantly from the true structure in terms of the relative position and orientation of the two trimers. Irrespective of such noticeable conformational difference, each predicted single trimer coincided well with the real trimer, as shown in Fig. 2 ▸(*c*).

### 2.5. Quality evaluation of the 3D reconstruction

In order to quantitatively measure the quality of the 3D reconstruction, multiple metrics were adopted in this study. CC shell: the correlation coefficients between the calculated 3D Fourier volume and the real volume at various resolution shells, is typically used to evaluate the resolution of the reconstructed Fourier volume. CC_mean_: the mean value of C (*n*, α_best_, β_best_, γ_best_) for all diffraction patterns at each iteration in the CM algorithm [see equation (5)], is used to monitor the convergence of the algorithm. CC map: the distribution of C (*n*, **R**, **γ_best_**) for a specified pattern across the entire orientation space{α, β} [see equation (4)], in which the degree of concentration reveals the level of fidelity of the CM algorithm. Angular deviation: the deviation between the calculated orientation and the real orientation for each diffraction pattern. Fourier shell correlation (FSC): the Fourier correlation coefficients between the recovered and the real electron-density maps as a function of resolution shell, which are used to evaluate the resolution of the recovered electron-density map arising from phase retrieval algorithm.

### 2.6. Computational environment

The algorithm was primarily implemented in C, utilizing MPI and OpenMP for parallelization to enhance computational efficiency. Python was employed for the analysis of the algorithm output. The computations were performed on a computer equipped with an Intel Core i7-12700 processor, comprising 12 cores and 20 threads. All computations were carried out on the CPU, without employing GPU resources. A single iteration of the orientation determination algorithm on 20 000 diffraction patterns took about 243 s. A single iteration of the phase retrieval algorithm was completed in approximately 1.9 s.

## 3. Results

### 3.1. Simulation validation of the predicted model-aided reconstruction method

To better understand the performance of the predicted model (PM)-aided reconstruction method, a total of 20 000 simulated diffraction patterns, with a photon count of 1 × 10^12^ per pulse, from the test protein 6zfp to assess the influence of the predicted model on the reconstruction process. In addition, the traditional reconstruction method starting from a randomly generated reference Fourier volume was also carried out for comparison (hereafter referred to as the random method).

**Figure 3:**
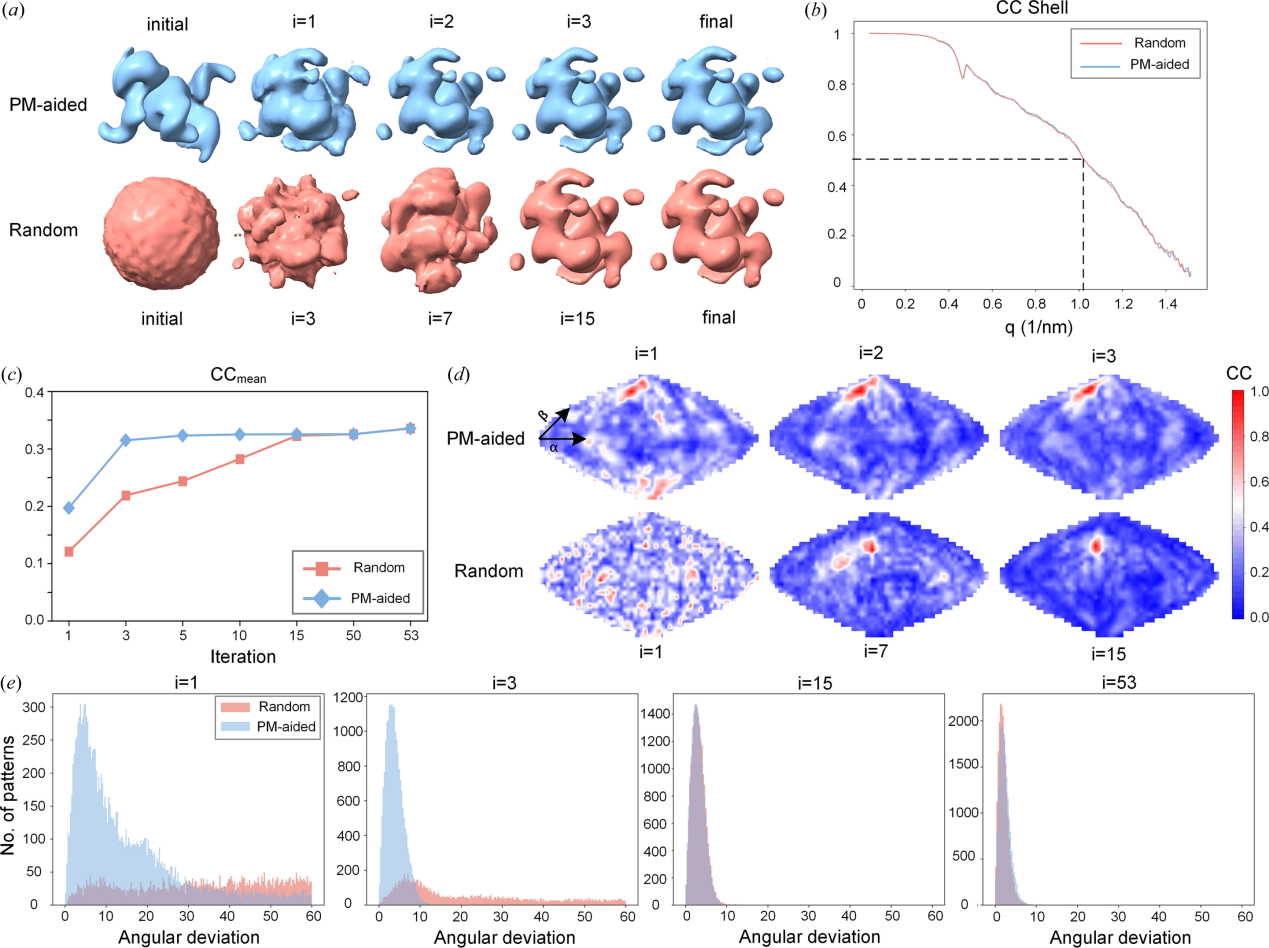
Comparison of the results from the PM-aided method and the random method in terms of orientation determination. Tests were conducted using the protein 6zfp. (*a*) 3D Fourier volumes at different iterations reconstructed by the PM-aided (blue) and random method (magenta). Note that the contour level is made identical for all volumes. (*b*) Plots of correlation coefficients between the real Fourier volume and the reconstructed Fourier volumes at various resolution shells for the PM-aided method (blue) and the random method (magenta). Note that the dotted line indicates the resolution of the reconstructed 3D Fourier volume, where the CC shell drops to 0.5. (*c*) Average of the maximum correlation coefficient between the calculated and experimental diffraction patterns (CC_mean_) as a function of iteration number for the PM-aided (blue) and random method (magenta). (*d*) CC map for a randomly selected diffraction pattern plotted in the elevation-azimuth plane at different iterations. The azimuth angle α is along the latitude direction while the elevation angle β is along the longitude direction. The upper row is calculated from the PM-aided method and the lower row is from the random method. The color scale is also shown. (*e*) Distribution of angular deviations at different iterations for the PM-aided method (blue) and the random method (magenta). Note that the units for angular deviation are given in degrees.

It can be observed that the reference Fourier volume yielded from the predicted model roughly resembles the overall profile of the actual Fourier volume, whereas the randomly generated Fourier volume appears as an amorphous sphere with no discernible features [Fig. 3 ▸(*a*)]. Expectedly, as the iterations progress, both reference Fourier volumes were able to converge to the final correct solution. However, the random method requires 15 iterations to reach full convergence while only three iterations are needed for the the PM-aided method, demonstrating that the PM-aided method could significantly accelerate the reconstruction procedure. To evaluate the resolution of each reconstructed Fourier volume, we employed CC shell to measure their similarities to the accurate Fourier volume at each resolution shell [Fig. 3 ▸(*b*)]. It can be clearly seen that both of them could achieve the same resolution of ∼10 Å. To quantitatively characterize the gradual improvement over the reconstruction process, CC_mean_ was calculated at each iteration for both methods. As shown in Fig. 3 ▸(*c*), both methods start at a relatively low value with a somewhat higher value for the PM-aided method, which is gradually increased to the same plateau within 53 iterations. It is likely that the PM-aided reconstruction rapidly converges with only 3 iterations, in sharp contrast to the random method requiring at least 15 iterations. Note that the slight increase at the last three iterations can be attributed to the refinement using finer sampling intervals. To further visualize the distribution of CC across the whole orientation space, we also plotted the CC map for a specified diffraction pattern at different cycles of iterations [Fig. 3 ▸(*d*)]. Clearly, for the random method, the initial map is filled with many spurious peaks, which gradually evolve into two major peaks at the 7th iteration and are ultimately consolidated into a single prominent peak at the 15th iteration. On the contrary, the initial map from the PM-aided method becomes considerably cleaner with fewer false peaks and only two rounds of iterations could distinguish the correct peak. Observations of the trajectory of the maximum values in the CC map after each iteration reveal that the changes in the optimal orientation are minimal in the PM-aided method (Video S1 of the supporting information). In contrast, the optimal orientation in the random method underwent significant changes before arriving at the correct result (Video S2 of the supporting information).

Moreover, the angular deviations for all diffraction patterns were calculated at each iteration to gain a better understanding about the accuracy of orientation determination [Fig. 3 ▸(*e*)]. For the first cycle of iteration, the angular deviations are distributed uniformly in a 60° range for the random method while biased towards 0° with a peak value at approximately 6° for the PM-aided method, indicating the importance of prior information for orientation determination. After three iterations, a relatively small peak around 8° suddenly emerges for the random method and the distribution for the PM-aided method becomes significantly narrow with a peak value at 3°, suggesting remarkable improvement for both methods. More iterations would have a negligible effect on the distribution of the PM-aided method, indicative of the convergence of orientation determination. However, the distribution for the random method is gradually driven up to 15 iterations. Benefiting from further refinement using a finer sampling interval, both distributions would simultaneously deflect toward a slightly lower angular deviation of 2°.

After orientation determination, the resulting 3D Fourier volume reconstructed with the PM-aided method was subsequently phased to generate the final electron-density map. The initial phase information either came from the predicted model or was directly set to random values. When employing random initial phases, the process involved 1000 iterations of the HIO algorithm, followed by 100 iterations of the ER algorithm. In contrast, when using predicted initial phases, only 1100 iterations of the ER algorithm were required. This distinction arises because the HIO algorithm, characterized by its exploratory nature, can broadly search for accurate results when initial values are imprecise. However, when initial values are close to the accurate result, the algorithm may inadvertently leap to incorrect regions, leading to erroneous outcomes. Tables S1–S3 of the supporting information include a detailed comparison of the two strategies under various conditions.

**Figure 4:**
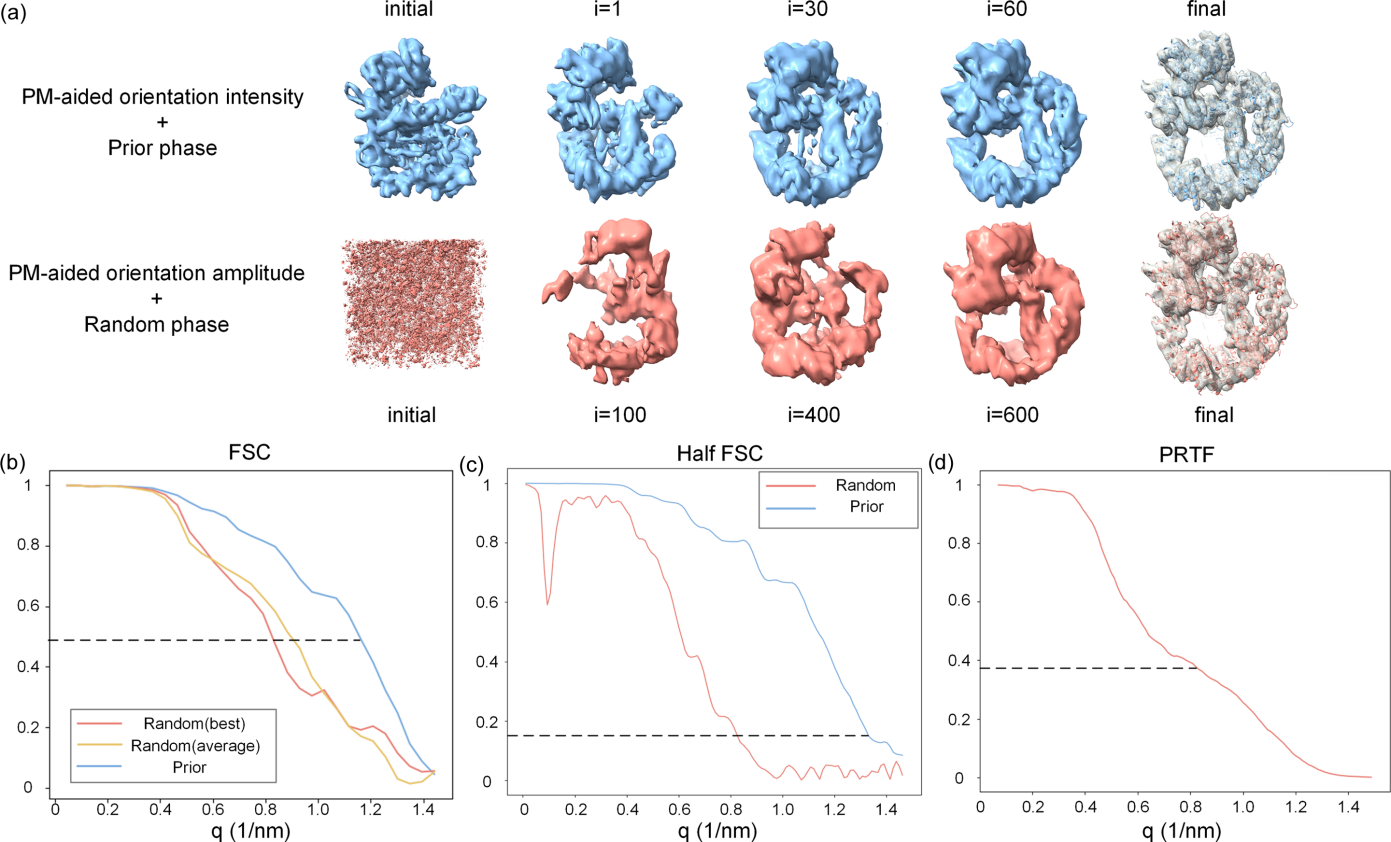
Comparison results of the phase retrieval using prior initial phases and random initial phases. Tests were conducted using the protein 6zfp. (*a*) Evolution of the electron-density maps at different iterations during the phase retrieval process. In the upper row, the initial map is calculated with phases of the predicted model. In the lower row, the initial map is calculated with random phases. The real protein structure is well docked into each recovered electron-density map. (*b*) FSC curve calculated by recover density and real density maps. The magenta and blue lines represent the recover density from the random initial phases and prior initial phases, respectively. The yellow line represents an average density of five recover densities from five independent random phases. The dotted line indicates the resolution of the reconstructed 3D electron-density map, where FSC drops to 0.5. (*c*) Half FSC curve. The dataset was randomly split into two sets of equal size. Orientation determination and phase retrieval were performed independently on each set. An FSC was then calculated based on the two resulting density maps. The magenta and blue lines represent recover density from random initial phases and prior initial phases, respectively. Note that the commonly used threshold in half FSC is 1.4. (*d*) Phase-retrieval transfer function from five successful recovered density maps from different random initial phases. The resolution is estimated by applying a threshold to the PRTF at e^−1^.

The evolution of the recovered electron-density map is comparatively depicted in Fig. 4 ▸(*a*). It can be observed that the initial map using prior phase information closely resembles the predicted model and is gradually developed into the actual structure within 60 cycles of iterations. In contrast, the electron-density map derived from random phases is initially chaotic, thus necessitating a substantially large amount of iterations to converge into the correct structure. Both recovered electron-density maps are of sufficiently high quality to accommodate the actual structure and the backbones can be accurately traced after rigid-body fitting. The FSC was calculated between the final recovered electron-density map and the real density map, with the results displayed in Fig. 4 ▸(*b*). Typically, a correlation coefficient threshold of 0.5 is used as a criterion for determining the resolution (Rosenthal & Henderson, 2003 ▸). According to this criterion, the resolution of the recovered map using prior phases (blue) is ∼8.7 Å, which is remarkably higher than the random phase (magenta) resolution of ∼12 Å. Furthermore, we performed phase retrieval using ten different sets of random initial phases, of which five were successful. Aligning and averaging the five successful recovered electron-density maps helped to average out noise and enhance the quality of the density map. The FSC of the averaged electron-density maps (yellow) indicates a resolution of ∼11 Å, which is slightly higher than that of a single map derived from random phases, but still much lower than the resolution obtained from the prior phase. Although using a greater number of random initial phases to obtain more density maps for averaging could further improve resolution, it would come at the cost of greatly increased computational time.

In real experiments, since the true electron-density map of the sample molecule is unknown, the resolution of the reconstructed electron densities is commonly assessed using half the FSC and phase retrieval transfer function (PRTF) (Latychevskaia, 2018 ▸; Shapiro *et al.*, 2005 ▸). To calculate the half FSC, we divided 20 000 diffraction patterns randomly into two sets of 10 000 patterns each and conducted orientation determination and phase retrieval separately for each set. The half FSC was then calculated using the two recovered electron-density maps, as shown in Fig. 4 ▸(*c*). Note that an FSC value of 0.5, calculated from the reconstructed density of the full dataset with the true density is equivalent to a half FSC of 0.143 (Rosenthal & Henderson, 2003 ▸). Based on the threshold of 0.143, the half FSC gives a resolution of ∼7.6 Å for the prior phases, also much higher than the ∼12 Å for random phases. For random initial phases, the phase retrieval transfer function was also calculated using electron densities from five different initial phases, as shown in Fig. 4 ▸(*d*). The resolution threshold in the PRTF is typically defined as e^−1^ (Chapman *et al.*, 2006*b* ▸), yielding a resolution of ∼12 Å.

Taken together, the introduction of a prior predicted model into the field of XFEL SPI markedly leverages the global convergence properties of both orientation determination and phase retrieval, substantially reducing the required number of iterations for convergence while simultaneously achieving a notably higher resolution in the recovered electron-density map.

### 3.2. Influence of different pulse fluences on orientation determination and phase retrieval

**Figure 5:**
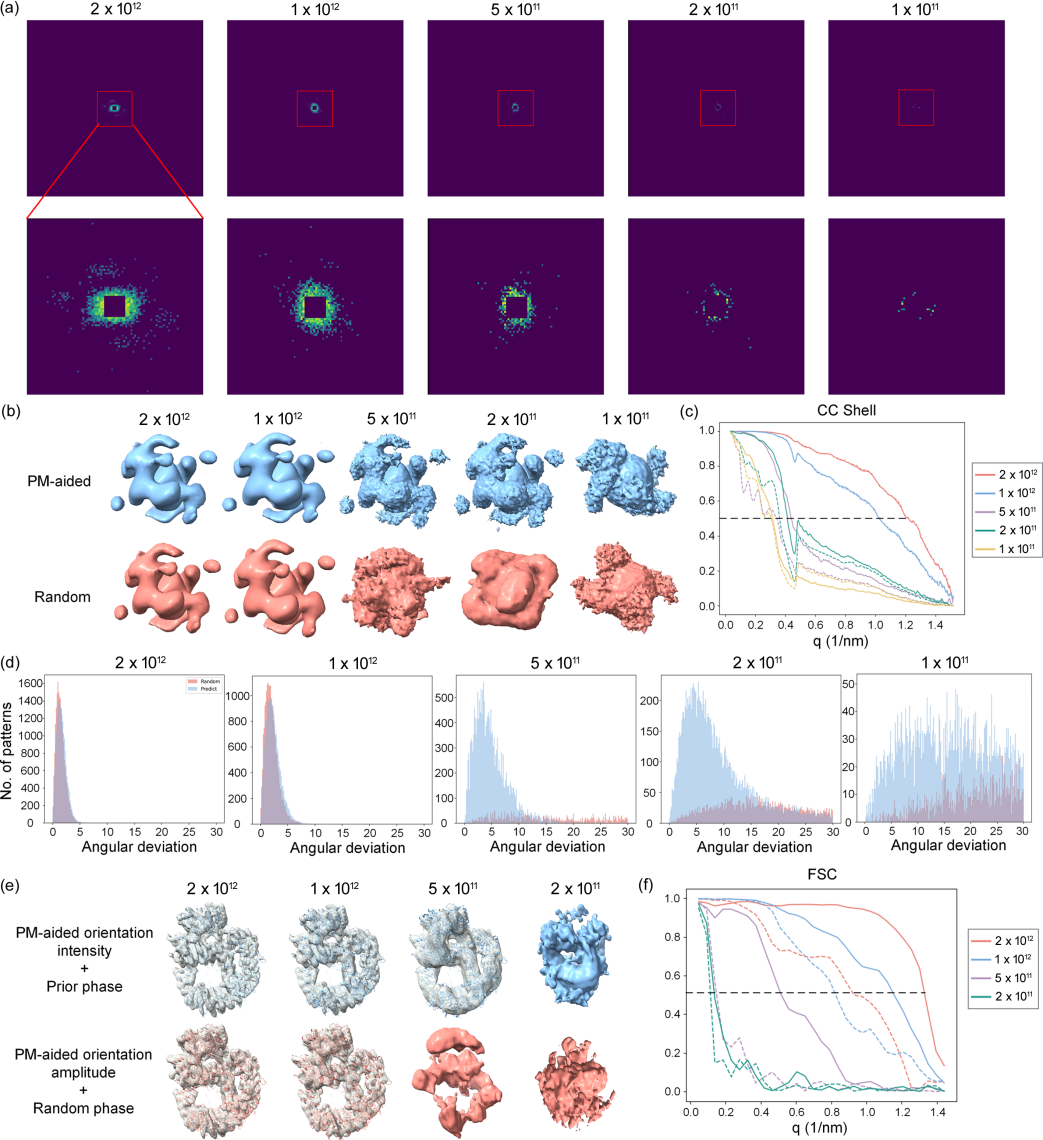
Influence of different pulse fluences on the orientation determination and phase retrieval. Tests were conducted using the protein 6zfp. (*a*) Diffraction pattern under different pulse fluences. The first line represents the full-size diffraction pattern (512 × 512), while the second line displays a cropped section of the center of the diffraction pattern (100 × 100). (*b*) Comparison of 3D Fourier volumes reconstructed by the PM-aided (blue) and random (magenta) methods at different pulse fluences. All volumes are rendered at the same contour level. (*c*) Comparison of both methods at different pulse fluences in terms of the CC shell. Solid lines represent the PM-aided method and dotted lines represent the random method. The horizontal line indicates the resolution of each reconstructed Fourier volume, where the CC shell is 0.5. (*d*) Comparison of the distribution of angular deviations at different pulse fluences for the PM-aided method (blue) and the random method (magenta). The units for angular deviations are given in degrees. (*e*) Comparison of electron-density maps after iterative phasing of the Fourier volumes reconstructed by the PM-aided method at different pulse fluences. In the upper row, the initial map is calculated with phases of the predicted model. In the lower row, the initial map is calculated with random phases. (*f*) Comparison of the FSCs of the recovered electron-density maps using prior phases (solid lines) and random phases (dotted lines) at various pulse fluences, plotted against the length of the scattering vector. The horizontal line indicates the resolution of the recovered 3D electron-density map, where FSC drops to 0.5.

One of the major bottlenecks in achieving high-resolution 3D reconstruction in SPI is due to the intrinsically low SNR of the diffraction patterns. Different pulse fluences lead to varying SNRs in the diffraction patterns, which will significantly impact the accuracy of orientation determination and the success rate of phase retrieval. In this section, we further evaluated the influence of pulse fluences on orientation determination and phase retrieval. The beam spot was set as a circle with a diameter of 0.1 µm, with the number of photons per pulse varying from 2 × 10^12^ to 1 × 10^11^. Fig. 5 ▸(*a*) displays diffraction patterns obtained at varying pulse fluences.

After applying the same reconstruction method to each set of diffraction patterns, the recovered 3D Fourier volumes for both the PM-aided method and the random method are comparatively shown in Fig. 5 ▸(*b*). When the pulse photon number is higher than 1 × 10^12^, both methods perform equally well, as shown by the visually identical Fourier volumes. However, the Fourier volumes deteriorated to diverse degrees for both methods at a pulse photon number below 1 × 10^12^. For the random method, the Fourier volume deviates significantly from the reference volume even at a pulse photon number of 5 × 10^11^. For the PM-aided method, the surface of the Fourier volume becomes somewhat coarser, while maintaining the correct morphology until the pulse photon number drops below 2 × 10^11^, indicating that the PM-aided method is more robust to low signal limit. The Fourier volume eventually collapses at a pulse photon number below 1 × 10^11^. This can be explained by the fact that the added noises at such low signal limit could not be smeared out using the given number of patterns, thus resulting in a worse Fourier volume which in turn decreases the accuracy of orientation determination. A similar conclusion can be drawn in terms of the CC shell [Fig. 5 ▸(*c*)] and the distribution of angular deviations [Fig. 5 ▸(*d*)]. When the pulse photon number is higher than 1 × 10^12^, both methods bring about the same results where higher pulse fluences could lead to more accurate orientations and achieve better resolution (∼8.3 Å for 2 × 10^12^ and ∼9.7 Å for 1 × 10^12^). However, when the pulse photon number falls below 1 × 10^12^, the CC shell of the PM-aided method demonstrates a significantly higher resolution (∼22 Å for 5 × 10^11^ and ∼25 Å for 2 × 10^11^) compared with its random counterpart (∼30 Å for 5 × 10^11^ and ∼29 Å for 2 × 10^11^), as shown in Fig. 5 ▸(*c*). Likewise, it can also be observed that the angular deviations for the PM-aided method are always subject to a skewed distribution with the peak value at ∼5° until the pulse photon number decreases to 1 × 10^11^ [Fig. 5 ▸(*d*)]. In contrast, the angular deviations for the random method are completely erroneous when the pulse photon number is lower than 1 × 10^12^, in line with the above observations.

Afterwards, the 3D Fourier volumes reconstructed with the PM-aided method at different pulse fluences were further phased using prior phases and random phases for comparison. In order to ensure a convincing result, we repeated the phase retrieval algorithm with different sets of random phases ten times and summarized the corresponding success rates. The test results are presented in Table 2 ▸, and the best recovered electron-density maps are depicted in Fig. 5 ▸(*e*). As expected, when the pulse photon number is higher than 1 × 10^12^, the Fourier volumes could be successfully phased with either phase set or the protein structure can be well docked into each recovered density map. However, when the pulse photon number declines to 5 × 10^11^, only the Fourier volume combined with prior phase information could be successfully phased while further reducing the pulse fluences will lead to utter failures. In parallel, the FSC curves also illustrate that the electron-density maps derived from prior phase information concurrently exhibit significantly higher resolutions than the corresponding maps recovered using initial random phases, as illustrated in Fig. 5 ▸(*f*). The resolutions of the electron-density maps are as follows: at a pulse photon number of 2 × 10^12^, the resolution is ∼7.5 Å for the prior phase and ∼11 Å for the random phase; at a pulse photon number of 1 × 10^12^, the resolution is ∼8.7 Å for the prior phase and ∼12 Å for the random phase. We also calculated the half FSC of the recovered electron-density map at different pulse fluences, yielding similar results, as shown in Fig. S1. This demonstrates that the use of prior phase information from the predicted model holds the promise of achieving better performance in structural determination, particularly at low signal levels.

### 3.3. Influence of different numbers of diffraction patterns on orientation determination and phase retrieval

The quality of 3D reconstruction depends heavily on the number of diffraction patterns, which has a tremendous impact on the convergence property of orientation determination. Increasing the number of diffraction patterns will decrease the noise in the assembled diffraction volume and *vice versa*. To this end, the influence of the number of diffraction patterns on orientation determination and phase retrieval was numerically studied using 20 000, 10 000, 5000, 1000 and 500 diffraction patterns, all simulated at the same pulse photon number of 1 × 10^12^.

**Figure 6:**
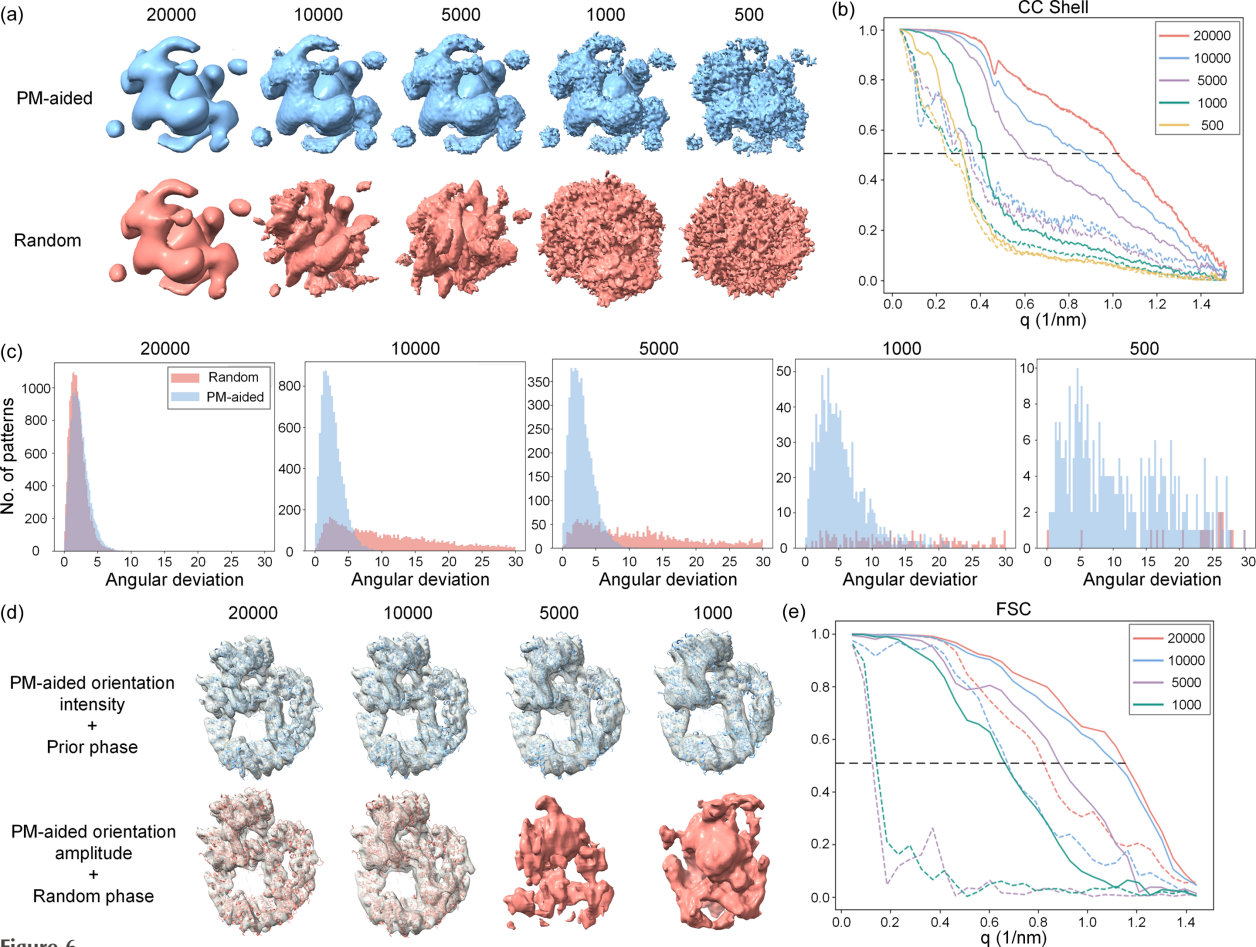
Influence of different numbers of diffraction patterns on the orientation determination and phase retrieval. Tests were conducted using the protein 6zfp. (*a*) Comparison of 3D Fourier volumes reconstructed by the PM-aided (blue) and random (magenta) methods using different numbers of diffraction patterns. All volumes are rendered at the same contour level. (*b*) Comparison of both methods using different numbers of patterns in terms of the CC shell. Solid lines represent the PM-aided method and dotted lines represent the random method. The horizontal line indicates the resolution of each reconstructed Fourier volume, where the CC shell is 0.5. (*c*) Comparison of the distribution of angular deviations using different numbers of diffraction patterns for the PM-aided method (blue) and the random method (magenta). The units for angular deviations are given in degrees. (*d*) Comparison of electron-density maps after iterative phasing of the Fourier volumes reconstructed by the PM-aided method under different numbers of patterns. In the upper row, the initial map is calculated with phases of the predicted model. In the lower row, the initial map is calculated with random phases. (*e*) Comparison of FSCs of the recovered electron-density maps using prior phases (solid lines) and random phases (dotted lines) under various numbers of patterns, plotted against the length of scattering vector. The horizontal line indicates the resolution of the recovered 3D electron-density map, where FSC drops to 0.5.

A comparison of each reconstructed Fourier volume generated from either the PM-aided method or the random method using different numbers of diffraction patterns is shown in Fig. 6 ▸(*a*). Obviously, as the pattern number declines, the Fourier volume from the PM-aided method becomes progressively worse with gradually degraded surface continuity or smoothness. This is because it becomes harder to average out noises or fill the complete reciprocal-space volume with fewer patterns, leading to artifacts in orientation determination. Nevertheless, the overall profile of the reconstructed volume remains generally unchanged even if the number is reduced to 500. Conversely, the Fourier volume from the random method deforms significantly as the number declines to 10 000 or less, possibly due to the relatively high noise levels in the patterns. The above observation is further quantified via a comparison of the CC shell for both methods [Fig. 6 ▸(*b*)]. There is no appreciable difference of CC shell between the two methods when the pattern number is beyond 20 000, whereas the largest gap occurs when the number of patterns halves. This gap always exists but becomes smaller as the number of patterns continues to decrease. The distribution of angular error is also compared in order to evaluate the accuracy of the orientation determination [Fig. 6 ▸(*c*)]. As can be seen, the majority of angular deviations for the PM-aided method are less than 10° under different conditions, gradually broadening as the number of patterns decreases. In contrast, the distribution of the random method only matches well with that of the PM-aided method when the number of patterns is greater than 20 000 but immediately flattens as the number declines to 10 000 or less.

As usual, the 3D Fourier volumes reconstructed with the PM-aided method under different conditions were further phased using prior phase information and random phases, respectively. As shown in Table 3 ▸, iterative phasing combined with prior phase information could successfully recover the correct electron-density map even when the number is reduced to 1000, whereas 10 000 patterns are needed to achieve convergence for the random phases with only 20% success rate. The recovered electron-density maps are comparatively shown in Fig. 6 ▸(*d*) and the resolution is evaluated based on the FSC shell [Fig. 6 ▸(*e*)]. Likewise, when the number of patterns exceeds 1000, all recovered electron-density maps starting from prior phase information can wrap around the protein structure tightly. Note that the recovered density map gradually loses some fine details as the number of patterns decreases, as showed by the significant reduction in resolution (from ∼8.5 Å for 20 000 patterns to ∼14 Å for 1000 patterns). On the contrary, only using at least 10 000 patterns can the protein structure be accurately docked into the recovered density maps derived from random phases at a resolution of ∼14 Å. We also calculated the half FSC of the recovered electron-density map using different numbers of diffraction patterns, yielding similar results, as shown in Fig. S2.

### 3.4. The effect of missing central data on phase retrieval

**Figure 7:**
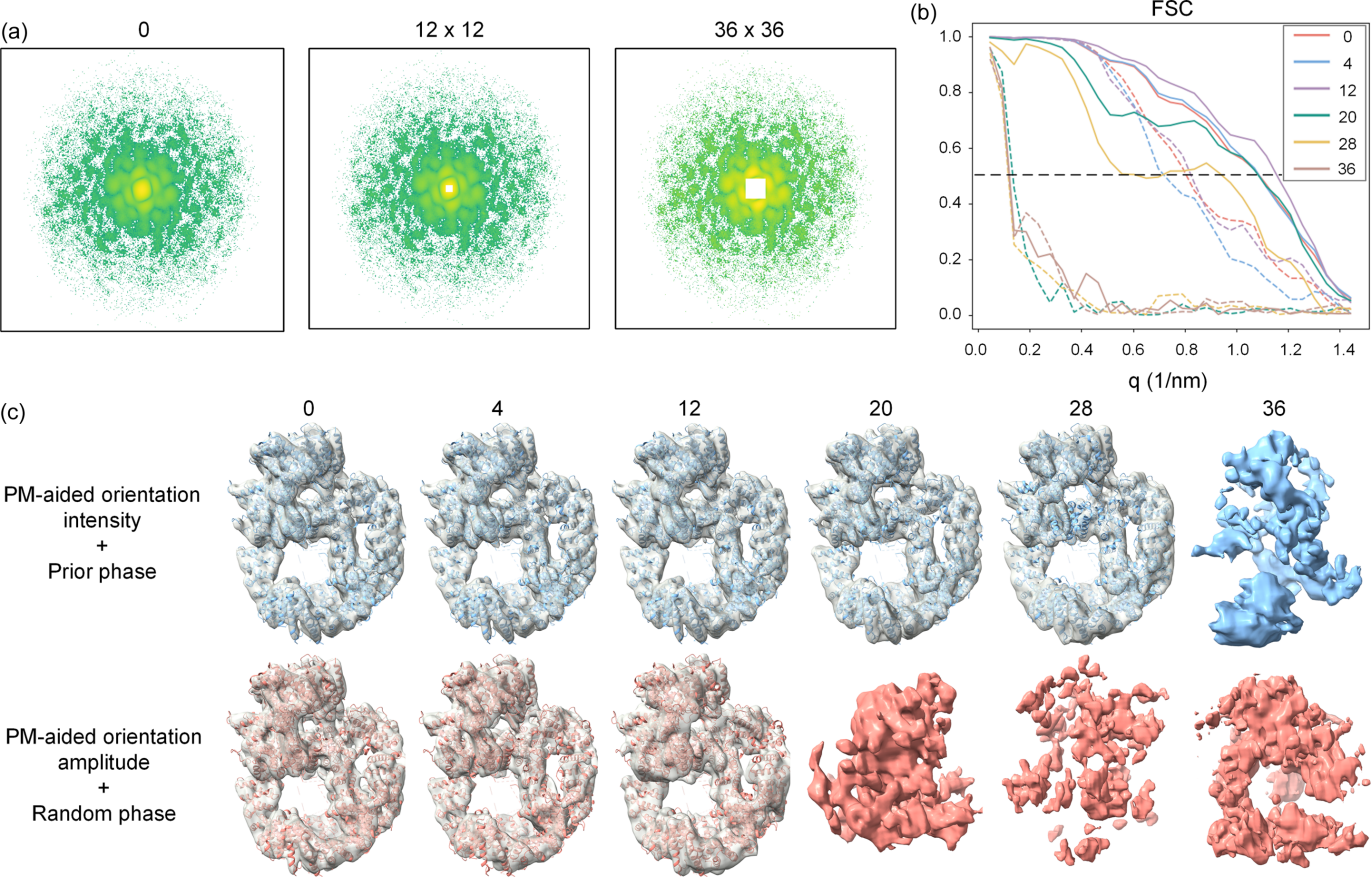
Effect of missing central data with different sizes on phase retrieval. Tests were conducted using the protein 6zfp. (*a*) Central slices of the reconstructed 3D Fourier volume with simulated beam stop of different sizes. The central white square represents the simulated beam stop. (*b*) Comparison of FSCs of the recovered electron-density maps using prior phases (solid lines) and random phases (dotted lines) under different sizes of simulated beam stop, plotted against the length of scattering vector. The horizontal line indicates the resolution of the recovered 3D electron-density map, where FSC drops to 0.5. (*c*) Comparison of electron-density maps after iterative phasing of the Fourier volumes with different sizes of missing central data. In the upper row, the initial map is calculated with phases of the predicted model. In the lower row, the initial map is calculated with random phases.

As mentioned above, the absence of low-resolution data poses a fundamental challenge to the success of phase retrieval. To assess the effect of missing low-resolution data on phase retrieval, we firstly simulated 20 000 diffraction patterns at a pulse photon number of 1 × 10^12^ without missing central data, followed by PM-aided orientation determination to generate a 3D Fourier volume. Subsequently, we manually removed the central data with various sizes from the reconstructed Fourier volume to simulate the presence of a beam stop. Of note, the size of the 3D volume is 512 × 512 × 512, and a beam stop size of 4 pixels indicates that the central 4 × 4 × 4 pixels in the 3D volume would be set to zero. For clarity, the maximum resolution corresponding to each size of beam stop is also listed in Table 4 ▸. Fig. 7 ▸(*a*) displays a visual comparison of central sections extracted from the 3D Fourier volume at different sizes of beam stop.

Routinely, iterative phasing of each volume in conjunction with either initial prior phases or initial random phases was applied to generate the real-space maps. The success rate of 3D phase recovery for each case is shown in Table 4 ▸. Clearly, the tolerable maximum resolution of the missing central data for successful phase retrieval is 56 nm using initial random phases, whereas the resolution limit is increased to 24 nm using initial prior phases. Moreover, the success rate of 3D phase recovery initiated with random phases is progressively decreased as the simulated size of the beam stop increases. The recovered electron-density maps are comparatively shown in Fig. 7 ▸(*c*) and the corresponding FSC shells are plotted in Fig. 7 ▸(*b*). In congruence with success rate, the ideal protein structure can be accurately fitted to the recovered maps initiated with prior phases until the resolution of the missing data reaches 24 nm, whereas it reduces to 56 nm for random phases. Above all, taking advantage of the prior phase information provided by the predicted model, the iterative phasing procedure can withstand significantly greater loss of central diffraction data.

### 3.5. Applicability for multimeric structure

Despite its spectacular structural prediction performance, structure prediction (*e.g.AlphaFold2*) is currently hampered by its limited ability to accurately model how multiple protein components assemble into functionally important integral machinery. To validate the applicability of the PM-aided method for multimeric structures, the test protein 7jpd comprising two trimers was adopted to simulate 20 000 diffraction patterns for study (see Table 1 ▸).

**Figure 8:**
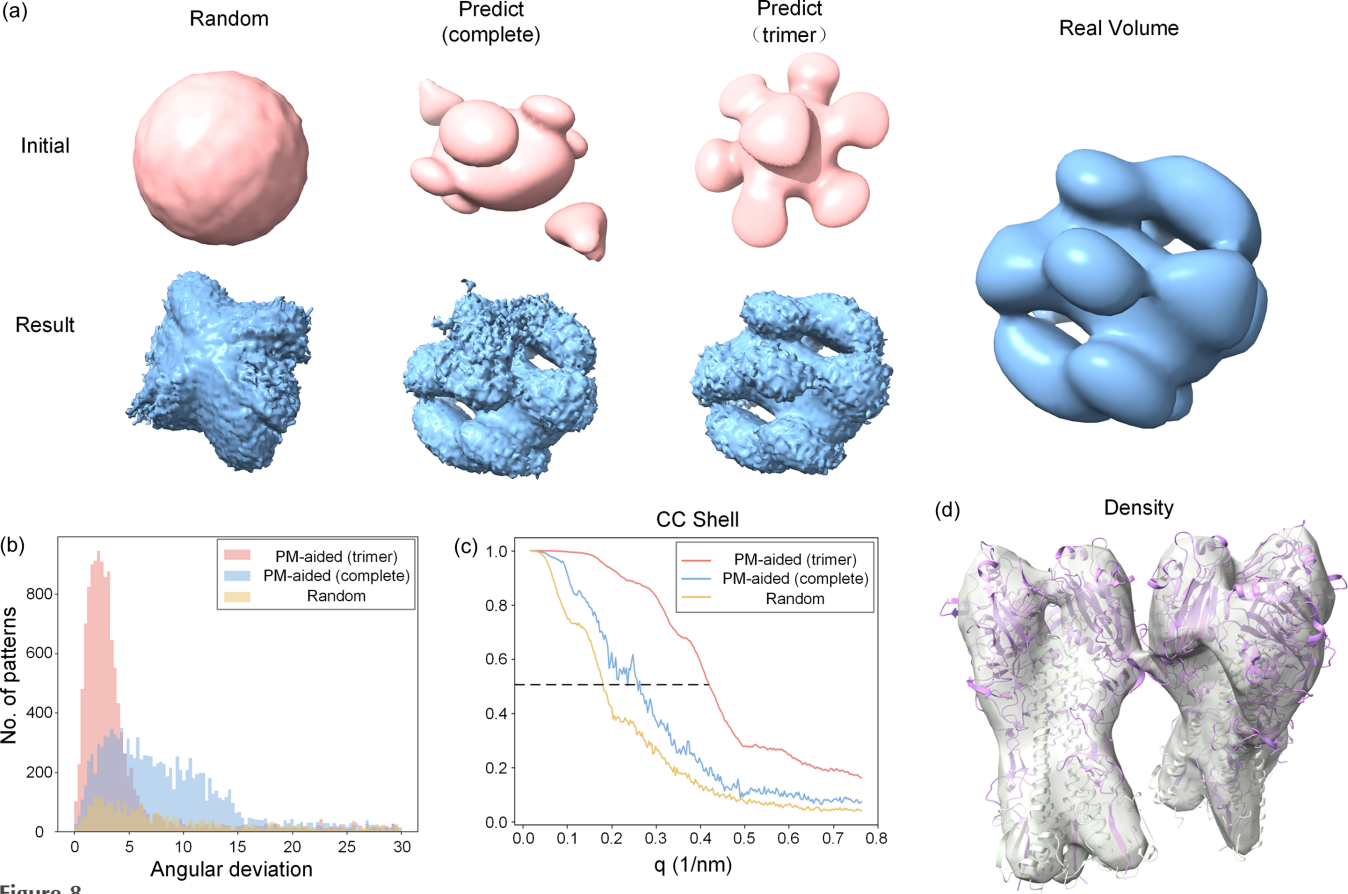
Application of the PM-aided method for the multimeric structure. Tests were conducted using the protein 7jpd. (*a*) Three reference Fourier volumes (upper row) used for orientation determination and the final reconstructed Fourier volumes (lower row). Each column represents the random volume (left), the volume of the two-trimer model (middle) and the volume of the single-trimer model (right). The ideal Fourier volume calculated from the real structure is also shown for comparison. (*b*) Comparison of the distribution of angular deviations for the complete model (blue), the single-trimer model (magenta) and the random model (yellow). The units for angular deviations are given in degrees. (*c*) CC shell between the resulting reciprocal-space intensity calculated with different initial conditions and the real intensity. (*d*) Recovered electron-density map derived from single-trimer model superimposed with the real protein structure.

Prior to orientation determination, we first examined different initial Fourier volumes generated separately from the predicted two-trimer model and the single-trimer model [Fig. 8 ▸(*a*)]. Obviously, both volumes significantly deviate from the ideal Fourier volume, with one volume shaped like a turbine (single-trimer model) and the other being fragile (two-trimer model). Using either volume as an initial reference volume for orientation determination could eventually give rise to a seemingly accurate Fourier volume [Fig. 8 ▸(*a*)]. Surprisingly, the reconstructed volume resulting from the single-trimer model bears a much closer resemblance to the ideal volume than that from the two-trimer model. It is supposed that the poorly assigned trimers relative to each other may account for such difference. By contrast, the randomly produced volume completely fails to converge into the correct solution in part due to the relatively low signal levels in the patterns. To further validate the above observations, some metrics such as angular deviation and CC shell are also displayed for comparison [Figs. 8 ▸(*b*) and 8 ▸(*c*)]. Obviously, most of angular deviations are less than 10° for the single-trimer model, resulting in the highest resolution of the reconstructed volume (∼22 Å). The distribution of angular errors quickly widens and extends to 15° for the two-trimer model, leading to a remarkable decrease in resolution (∼33 Å). As expected, the worst case occurs for the random volume where most angular deviations are distributed uniformly within a range of 180°, indicating the failure of orientation determination. Based on both successfully reconstructed Fourier volumes, iterative phasing with prior phase information from each predicted model was further carried out. Strikingly, only the Fourier volume reconstructed from the single-trimer model can be successfully phased [Fig. 8 ▸(*d*)]. It is assumed that the highly noisy Fourier volume reconstructed from the two-trimer model might prevent the phase recovery procedure. In summary, our results demonstrate that the PM-aided method is also applicable to multimeric structures.

## 4. Discussion and conclusions

Our series of tests demonstrated that a predicted protein structure can serve as a more effective starting point for orientation determination and phase retrieval compared with unpredictable random orientations and phases. This is primarily evident in the following ways. (1) It significantly accelerates the convergence of orientation determination and phase retrieval algorithms. (2) It could be applicable in situations with lower SNR and fewer diffraction patterns. (3) It effectively assists in overcoming the impact of missing low-resolution data on phase retrieval.

Furthermore, simulation experiments on multimers suggest that utilizing partial predicted structures as templates can also effectively assist in orientation determination. However, to utilize partial predicted structures for calculating initial phases, a ‘molecular replacement’ process is necessary. This involves computing multiple copies of a partial structure and positioning them to align the initial phases with the computed amplitudes. Due to the differences between SPI and crystallography, existing molecular replacement methods need to be specifically adapted or a novel approach tailored for single-molecule imaging should be developed.

Symmetry is frequently observed in multimer protein molecules. Effectively utilizing the inherent symmetry in protein molecules can significantly reduce the search space during orientation determination. This implies that fewer angles are required when sampling the orientation of 3D Fourier intensities, with the addition of symmetry sufficing to cover the entire 3D space. Each diffraction pattern only needs to be compared with fewer 2D slices. This paper does not utilize the conveniences afforded by symmetry. However, future research should consider how to better utilize symmetry to improve the computational efficiency of orientation determination.

Note that the diffraction signal from single molecules is significantly weaker than that from crystals. Therefore, current XFEL-based SPI experiments are limited to high-molecular-weight proteins and virus particles, which can usually be resolved using cryo-electron microscopy. Additionally, the resolution of structures derived from XFEL single-particle experiments remains considerably low. This technique is still in the early stages of exploration and feasibility verification and has yet to outperform cryo-EM under current experimental conditions. However, as XFEL sources continue to improve and pulse photon number increases, it will become possible to achieve diffraction imaging of smaller protein molecules with enhanced resolution. At that time, the unique conditions of XFEL, including imaging at ambient temperatures and pressures and the use of femtosecond pulses, will then offer distinct advantages for structural studies of protein physiological states and for conducting time-resolved analyses.

In addition, classification of diffraction patterns has long been a challenge in the process of single-molecule imaging data analysis. Ongoing research in our team has revealed that the introduction of predicted models can greatly assist in addressing these classification issues.

We have open-sourced our code. All code used in this article can be found at https://github.com/ZhichaoJiao/SPI_reconstruction.git

## Supplementary Material

Supporting movie 1. DOI: 10.1107/S2052252524004858/it5035sup1.avi

Supporting movie 2. DOI: 10.1107/S2052252524004858/it5035sup2.avi

Supporting figures and tables. DOI: 10.1107/S2052252524004858/it5035sup3.pdf

## Figures and Tables

**Table 1:** Parameters used in the simulation of diffraction patterns

	PDB entry 6zfp	PDB entry 7jpd
No. of amino acid residues	3704	2895
Molecular weight (kDa)	472.06	357.22
XFEL wavelength (Å)	1	1
No. of photons per pulse	1 × 10^11^ – 2 × 10^12^	1 × 10^12^
Beam focus size (µm)	0.1	0.1
Detector size (pixels)	512 × 512	512 × 512
Pixel size (µm)	300	300
Beam stop size (pixels)	0 × 0–36 × 36	12 × 12
Sample-to-detector distance (m)	0.5	1
Limit resolution at the edge of the pattern (Å)	6.6	13.1
No. of patterns	250–20000	20000

**Table 2:** Success rates of iterative phasing using prior phases and random phases under different pulse fluences The success of phase recovery is determined by visual inspection of the reconstructed electron-density maps, as well as by calculating the overall correlation coefficient between the reconstructed and the true electron-density maps. The overall correlation coefficient under different pulse fluences is shown in Fig. S4.

No. of photons per pulse	Beam focus size (µm)	No. of photons per pattern (avearge)	Prior phases	Random phases
2 × 10^12^	0.1	4400	1/1	5/10
1 × 10^12^	0.1	2400	1/1	5/10
5 × 10^11^	0.1	1100	1/1	0/10
2 × 10^11^	0.1	400	0/1	0/10

**Table 3:** Success rates of iterative phasing using prior phases and random phases with different numbers of diffraction patterns The success of phase recovery is determined by visual inspection of the reconstructed electron-density maps, as well as by calculating the overall correlation coefficient between the reconstructed and the true electron-density maps. The overall correlation coefficient using different number of patterns is shown in Fig. S5.

No. of patterns	Prior phases	Random phases
20000	1/1	5/10
10000	1/1	2/10
5000	1/1	0/10
1000	1/1	0/10
500	0/1	0/10

**Table 4:** Success rates of iterative phasing using prior phases and random phases under different sizes of beam stop The success of phase recovery is determined by visual inspection of the reconstructed electron-density maps, as well as by calculating the overall correlation coefficient between the reconstructed and true electron-density maps. The overall correlation coefficient under different size of beam stop is shown in Fig. S6.

Beam stop size (pixels)	Resolution (nm)	Prior phases	Random phases
0	∞	1/1	10/10
4	167	1/1	8/10
12	56	1/1	5/10
20	33	1/1	0/10
28	24	1/1	0/10
36	19	0/1	0/10

## References

[bb1] Assalauova, D., Kim, Y. Y., Bobkov, S., Khubbutdinov, R., Rose, M., Alvarez, R., Andreasson, J., Balaur, E., Contreras, A., DeMirci, H., Gelisio, L., Hajdu, J., Hunter, M. S., Kurta, R. P., Li, H., McFadden, M., Nazari, R., Schwander, P., Teslyuk, A., Walter, P., Xavier, P. L., Yoon, C. H., Zaare, S., Ilyin, V. A., Kirian, R. A., Hogue, B. G., Aquila, A. & Vartanyants, I. A. (2020). *IUCrJ*, **7**, 1102–1113. 10.1107/S2052252520012798 PMC7642788 33209321

[bb2] Ayyer, K., Lan, T.-Y., Elser, V. & Loh, N. D. (2016). *J. Appl. Cryst.* **49**, 1320–1335. 10.1107/S1600576716008165 PMC4970497 27504078

[bb3] Ayyer, K., Morgan, A. J., Aquila, A., DeMirci, H., Hogue, B. G., Kirian, R. A., Xavier, P. L., Yoon, C. H., Chapman, H. N. & Barty, A. (2019). *Opt. Express*, **27**, 37816–37833. 10.1364/OE.27.037816 31878556

[bb4] Baker, L. A. & Rubinstein, J. L. (2010). *Methods Enzymol.* **481**, 371–388. 10.1016/S0076-6879(10)81015-8 20887865

[bb5] Bock, L. V. & Grubmüller, H. (2022). *Nat. Commun.* **13**, 1709. 10.1038/s41467-022-29332-2 PMC8971465 35361752

[bb6] Bortel, G., Faigel, G. & Tegze, M. (2009). *J. Struct. Biol.* **166**, 226–233. 10.1016/j.jsb.2009.01.005 19374022

[bb7] Bortel, G. & Tegze, M. (2011). *Acta Cryst.* A**67**, 533–543. 10.1107/S0108767311036269 22011469

[bb8] Brink, J., Ludtke, S. J., Kong, Y., Wakil, S. J., Ma, J. & Chiu, W. (2004). *Structure*, **12**, 185–191. 10.1016/j.str.2004.01.015 14962379

[bb9] Chaplin, A. K., Hardwick, S. W., Liang, S., Kefala Stavridi, A., Hnizda, A., Cooper, L. R., De Oliveira, T. M., Chirgadze, D. Y. & Blundell, T. L. (2021). *Nat. Struct. Mol. Biol.* **28**, 13–19. 10.1038/s41594-020-00517-x 33077952

[bb10] Chapman, H. N., Barty, A., Bogan, M. J., Boutet, S., Frank, M., Hau-Riege, S. P., Marchesini, S., Woods, B. W., Bajt, S., Benner, H., London, R. A., Plönjes, E., Kuhlmann, M., Treusch, R., Düsterer, S., Tschentscher, T., Schneider, J. R., Spiller, E., Möller, T., Bostedt, C., Hoener, M., Shapiro, D. A., Hodgson, K. O., van der Spoel, D., Burmeister, F., Bergh, M., Caleman, C., Huldt, G., Seibert, M. M., Maia, F. R. N. C., Lee, R. W., Szöke, A., Timneanu, N. & Hajdu, J. (2006*a*). *Nat. Phys.* **2**, 839–843.

[bb12] Chapman, H. N., Barty, A., Marchesini, S., Noy, A., Hau-Riege, S. R., Cui, C., Howells, M. R., Rosen, R., He, H., Spence, J. C. H., Weierstall, U., Beetz, T., Jacobsen, C. & Shapiro, D. (2006*b*). *J. Opt. Soc. Am. A*, **23**, 1179–1200. 10.1364/josaa.23.001179 16642197

[bb13] Cheng, Y., Grigorieff, N., Penczek, P. A. & Walz, T. (2015). *Cell*, **161**, 438–449. 10.1016/j.cell.2015.03.050 PMC4409659 25910204

[bb14] Decking, W., Abeghyan, S., Abramian, P., Abramsky, A., Aguirre, A., Albrecht, C., Alou, P., Altarelli, M., Altmann, P., Amyan, K., Anashin, V., Apostolov, E., Appel, K., Auguste, D., Ayvazyan, V., Baark, S., Babies, F., Baboi, N., Bak, P., Balandin, V., Baldinger, R., Baranasic, B., Barbanotti, S., Belikov, O., Belokurov, V., Belova, L., Belyakov, V., Berry, S., Bertucci, M., Beutner, B., Block, A., Blöcher, M., Böckmann, T., Bohm, C., Böhnert, M., Bondar, V., Bondarchuk, E., Bonezzi, M., Borowiec, P., Bösch, C., Bösenberg, U., Bosotti, A., Böspflug, R., Bousonville, M., Boyd, E., Bozhko, Y., Brand, A., Branlard, J., Briechle, S., Brinker, F., Brinker, S., Brinkmann, R., Brockhauser, S., Brovko, O., Brück, H., Brüdgam, A., Butkowski, L., Büttner, T., Calero, J., Castro-Carballo, E., Cattalanotto, G., Charrier, J., Chen, J., Cherepenko, A., Cheskidov, V., Chiodini, M., Chong, A., Choroba, S., Chorowski, M., Churanov, D., Cichalewski, W., Clausen, M., Clement, W., Cloué, C., Cobos, J. A., Coppola, N., Cunis, S., Czuba, K., Czwalinna, M., D’Almagne, B., Dammann, J., Danared, H., de Zubiaurre Wagner, A., Delfs, A., Delfs, T., Dietrich, F., Dietrich, T., Dohlus, M., Dommach, M., Donat, A., Dong, X., Doynikov, N., Dressel, M., Duda, M., Duda, P., Eckoldt, H., Ehsan, W., Eidam, J., Eints, F., Engling, C., Englisch, U., Ermakov, A., Escherich, K., Eschke, J., Saldin, E., Faesing, M., Fallou, A., Felber, M., Fenner, M., Fernandes, B., Fernández, J. M., Feuker, S., Filippakopoulos, K., Floettmann, K., Fogel, V., Fontaine, M., Francés, A., Martin, I. F., Freund, W., Freyermuth, T., Friedland, M., Fröhlich, L., Fusetti, M., Fydrych, J., Gallas, A., García, O., Garcia-Tabares, L., Geloni, G., Gerasimova, N., Gerth, C., Geßler, P., Gharibyan, V., Gloor, M., Głowinkowski, J., Goessel, A., Gołębiewski, Z., Golubeva, N., Grabowski, W., Graeff, W., Grebentsov, A., Grecki, M., Grevsmuehl, T., Gross, M., Grosse-Wortmann, U., Grünert, J., Grunewald, S., Grzegory, P., Feng, G., Guler, H., Gusev, G., Gutierrez, J. L., Hagge, L., Hamberg, M., Hanneken, R., Harms, E., Hartl, I., Hauberg, A., Hauf, S., Hauschildt, J., Hauser, J., Havlicek, J., Hedqvist, A., Heidbrook, N., Hellberg, F., Henning, D., Hensler, O., Hermann, T., Hidvégi, A., Hierholzer, M., Hintz, H., Hoffmann, F., Hoffmann, M., Hoffmann, M., Holler, Y., Hüning, M., Ignatenko, A., Ilchen, M., Iluk, A., Iversen, J., Iversen, J., Izquierdo, M., Jachmann, L., Jardon, N., Jastrow, U., Jensch, K., Jensen, J., Jeżabek, M., Jidda, M., Jin, H., Johansson, N., Jonas, R., Kaabi, W., Kaefer, D., Kammering, R., Kapitza, H., Karabekyan, S., Karstensen, S., Kasprzak, K., Katalev, V., Keese, D., Keil, B., Kholopov, M., Killenberger, M., Kitaev, B., Klimchenko, Y., Klos, R., Knebel, L., Koch, A., Koepke, M., Köhler, S., Köhler, W., Kohlstrunk, N., Konopkova, Z., Konstantinov, A., Kook, W., Koprek, W., Körfer, M., Korth, O., Kosarev, A., Kosiński, K., Kostin, D., Kot, Y., Kotarba, A., Kozak, T., Kozak, V., Kramert, R., Krasilnikov, M., Krasnov, A., Krause, B., Kravchuk, L., Krebs, O., Kretschmer, R., Kreutzkamp, J., Kröplin, O., Krzysik, K., Kube, G., Kuehn, H., Kujala, N., Kulikov, V., Kuzminych, V., La Civita, D., Lacroix, M., Lamb, T., Lancetov, A., Larsson, M., Le Pinvidic, D., Lederer, S., Lensch, T., Lenz, D., Leuschner, A., Levenhagen, F., Li, Y., Liebing, J., Lilje, L., Limberg, T., Lipka, D., List, B., Liu, J., Liu, S., Lorbeer, B., Lorkiewicz, J., Lu, H. H., Ludwig, F., Machau, K., Maciocha, W., Madec, C., Magueur, C., Maiano, C., Maksimova, I., Malcher, K., Maltezopoulos, T., Mamoshkina, E., Manschwetus, B., Marcellini, F., Marinkovic, G., Martinez, T., Martirosyan, H., Maschmann, W., Maslov, M., Matheisen, A., Mavric, U., Meißner, J., Meissner, K., Messerschmidt, M., Meyners, N., Michalski, G., Michelato, P., Mildner, N., Moe, M., Moglia, F., Mohr, C., Mohr, S., Möller, W., Mommerz, M., Monaco, L., Montiel, C., Moretti, M., Morozov, I., Morozov, P., Mross, D., Mueller, J., Müller, C., Müller, J., Müller, K., Munilla, J., Münnich, A., Muratov, V., Napoly, O., Näser, B., Nefedov, N., Neumann, R., Neumann, R., Ngada, N., Noelle, D., Obier, F., Okunev, I., Oliver, J. A., Omet, M., Oppelt, A., Ottmar, A., Oublaid, M., Pagani, C., Paparella, R., Paramonov, V., Peitzmann, C., Penning, J., Perus, A., Peters, F., Petersen, B., Petrov, A., Petrov, I., Pfeiffer, S., Pflüger, J., Philipp, S., Pienaud, Y., Pierini, P., Pivovarov, S., Planas, M., Pławski, E., Pohl, M., Polinski, J., Popov, V., Prat, S., Prenting, J., Priebe, G., Pryschelski, H., Przygoda, K., Pyata, E., Racky, B., Rathjen, A., Ratuschni, W., Regnaud-Campderros, S., Rehlich, K., Reschke, D., Robson, C., Roever, J., Roggli, M., Rothenburg, J., Rusiński, E., Rybaniec, R., Sahling, H., Salmani, M., Samoylova, L., Sanzone, D., Saretzki, F., Sawlanski, O., Schaffran, J., Schlarb, H., Schlösser, M., Schlott, V., Schmidt, C., Schmidt-Foehre, F., Schmitz, M., Schmökel, M., Schnautz, T., Schneidmiller, E., Scholz, M., Schöneburg, B., Schultze, J., Schulz, C., Schwarz, A., Sekutowicz, J., Sellmann, D., Semenov, E., Serkez, S., Sertore, D., Shehzad, N., Shemarykin, P., Shi, L., Sienkiewicz, M., Sikora, D., Sikorski, M., Silenzi, A., Simon, C., Singer, W., Singer, X., Sinn, H., Sinram, K., Skvorodnev, N., Smirnow, P., Sommer, T., Sorokin, A., Stadler, M., Steckel, M., Steffen, B., Steinhau-Kühl, N., Stephan, F., Stodulski, M., Stolper, M., Sulimov, A., Susen, R., Świerblewski, J., Sydlo, C., Syresin, E., Sytchev, V., Szuba, J., Tesch, N., Thie, J., Thiebault, A., Tiedtke, K., Tischhauser, D., Tolkiehn, J., Tomin, S., Tonisch, F., Toral, F., Torbin, I., Trapp, A., Treyer, D., Trowitzsch, G., Trublet, T., Tschentscher, T., Ullrich, F., Vannoni, M., Varela, P., Varghese, G., Vashchenko, G., Vasic, M., Vazquez-Velez, C., Verguet, A., Vilcins-Czvitkovits, S., Villanueva, R., Visentin, B., Viti, M., Vogel, E., Volobuev, E., Wagner, R., Walker, N., Wamsat, T., Weddig, H., Weichert, G., Weise, H., Wenndorf, R., Werner, M., Wichmann, R., Wiebers, C., Wiencek, M., Wilksen, T., Will, I., Winkelmann, L., Winkowski, M., Wittenburg, K., Witzig, A., Wlk, P., Wohlenberg, T., Wojciechowski, M., Wolff-Fabris, F., Wrochna, G., Wrona, K., Yakopov, M., Yang, B., Yang, F., Yurkov, M., Zagorodnov, I., Zalden, P., Zavadtsev, A., Zavadtsev, D., Zhirnov, A., Zhukov, A., Ziemann, V., Zolotov, A., Zolotukhina, N., Zummack, F. & Zybin, D. (2020). *Nat. Photon.* **14**, 391–397.

[bb15] DePonte, D. P., Weierstall, U., Schmidt, K., Warner, J., Starodub, D., Spence, J. C. H. & Doak, R. B. (2008). *J. Phys. D Appl. Phys.* **41**, 195505.

[bb16] Ekeberg, T., Assalauova, D., Bielecki, J., Boll, R., Daurer, B. J., Eichacker, L. A., Franken, L. E., Galli, D. E., Gelisio, L., Gumprecht, L., Gunn, L. H., Hajdu, J., Hartmann, R., Hasse, D., Ignatenko, A., Koliyadu, J., Kulyk, O., Kurta, R., Kuster, M., Lugmayr, W., Lübke, J., Mancuso, A. P., Mazza, T., Nettelblad, C., Ovcharenko, Y., Rivas, D. E., Samanta, A. K., Schmidt, P., Sobolev, E., Timneanu, N., Usenko, S., Westphal, D., Wollweber, T., Worbs, L., Xavier, P. L., Yousef, H., Ayyer, K., Chapman, H. N., Sellberg, J. A., Seuring, C., Vartanyants, I. A., Küpper, J., Meyer, M. & Maia, F. R. N. C. (2022). *bioRxiv*:2022.03.09.483477.

[bb17] Ekeberg, T., Svenda, M., Abergel, C., Maia, F. R., Seltzer, V., Claverie, J., Hantke, M., Jönsson, O., Nettelblad, C., van der Schot, G., Liang, M., DePonte, D. P., Barty, A., Seibert, M. M., Iwan, B., Andersson, I., Loh, N. D., Martin, A. V., Chapman, H., Bostedt, C., Bozek, J. D., Ferguson, K. R., Krzywinski, J., Epp, S. W., Rolles, D., Rudenko, A., Hartmann, R., Kimmel, N. & Hajdu, J. (2015). *Phys. Rev. Lett.* **114**, 098102. 10.1103/PhysRevLett.114.098102 25793853

[bb18] Fessler, J. A. & Sutton, B. P. (2003). *IEEE Trans. Signal Process.* **51**, 560–574.

[bb19] Fienup, J. R. (1982). *Appl. Opt.* **21**, 2758–2769. 10.1364/AO.21.002758 20396114

[bb20] Fowler, N. J. & Williamson, M. P. (2022). *Structure*, **30**, 925–933.e2. 10.1016/j.str.2022.04.005 PMC9592556 35537451

[bb21] Fung, R., Shneerson, V., Saldin, D. K. & Ourmazd, A. (2008). *Nat. Phys.* **5**, 64–67.

[bb22] Gaffney, K. J. & Chapman, H. N. (2007). *Science*, **316**, 1444–1448. 10.1126/science.1135923 17556577

[bb23] Gao, H., Valle, M., Ehrenberg, M. & Frank, J. (2004). *J. Struct. Biol.* **147**, 283–290. 10.1016/j.jsb.2004.02.008 15450297

[bb24] Garman, E. F. (2014). *Science*, **343**, 1102–1108. 10.1126/science.1247829 24604194

[bb25] Geng, Z., She, Z., Zhou, Q., Dong, Z., Zhan, F., Zhang, H., Xu, J., Gao, Z. & Dong, Y. (2021). *J. Struct. Biol.* **213**, 107770. 10.1016/j.jsb.2021.107770 34303831

[bb26] Ghafoori, S. M., Petersen, G. F., Conrady, D. G., Calhoun, B. M., Stigliano, M. Z. Z., Baydo, R. O., Grice, R., Abendroth, J., Lorimer, D. D., Edwards, T. E. & Forwood, J. K. (2023). *Sci. Rep.* **13**, 6940. 10.1038/s41598-023-33529-w PMC10140725 37117205

[bb27] Glaeser, R. M. (2016). *Methods Enzymol.* **579**, 19–50. 10.1016/bs.mie.2016.04.010 27572722

[bb28] Grigorieff, N. (2007). *J. Struct. Biol.* **157**, 117–125. 10.1016/j.jsb.2006.05.004 16828314

[bb29] Hantke, M. F., Hasse, D., Maia, F. R. N. C., Ekeberg, T., John, K., Svenda, M., Loh, N. D., Martin, A. V., Timneanu, N., Larsson, D. S. D., van der Schot, G., Carlsson, G. H., Ingelman, M., Andreasson, J., Westphal, D., Liang, M. N., Stellato, F., DePonte, D. P., Hartmann, R., Kimmel, N., Kirian, R. A., Seibert, M. M., Mühlig, K., Schorb, S., Ferguson, K., Bostedt, C., Carron, S., Bozek, J. D., Rolles, D., Rudenko, A., Epp, S., Chapman, H. N., Barty, A., Hajdu, J. & Andersson, I. (2014). *Nat. Photon.* **8**, 943–949.

[bb30] Harauz, G. & Ottensmeyer, F. P. (1983). *Ultramicroscopy*, **12**, 309–319. 10.1016/0304-3991(83)90305-4 6659150

[bb31] Harauz, G. & Ottensmeyer, F. P. (1984). *Science*, **226**, 936–940. 10.1126/science.6505674 6505674

[bb32] Heel, M. van (1984). *Ultramicroscopy*, **13**, 165–183. 10.1016/0304-3991(84)90066-4 6382731

[bb33] Heymann, J. B., Conway, J. F. & Steven, A. C. (2004). *J. Struct. Biol.* **147**, 291–301. 10.1016/j.jsb.2004.02.006 15450298

[bb34] Hosseinizadeh, A., Mashayekhi, G., Copperman, J., Schwander, P., Dashti, A., Sepehr, R., Fung, R., Schmidt, M., Yoon, C. H., Hogue, B. G., Williams, G. J., Aquila, A. & Ourmazd, A. (2017). *Nat. Methods*, **14**, 877–881. 10.1038/nmeth.4395 28805793

[bb35] Hryc, C. F. & Baker, M. L. (2022). *iScience* **25**, 104496. 10.1016/j.isci.2022.104496 PMC9207676 35733789

[bb36] Hu, L., Salmen, W., Sankaran, B., Lasanajak, Y., Smith, D. F., Crawford, S. E., Estes, M. K. & Prasad, B. V. V. (2022). *Commun. Biol.* **5**, 419. 10.1038/s42003-022-03357-1 PMC9072675 35513489

[bb37] Jiang, Y.-M., Miao, H., Pan, X.-Y., Wang, Q., Dong, Z., Geng, Z. & Dong, Y.-H. (2023). *Acta Cryst.* D**79**, 610–623. 10.1107/S2059798323004412 37314408

[bb38] Jorda, J., Sawaya, M. R. & Yeates, T. O. (2016). *Acta Cryst.* D**72**, 446–453. 10.1107/S2059798316003405 PMC4784676 26960132

[bb39] Jumper, J., Evans, R., Pritzel, A., Green, T., Figurnov, M., Ronneberger, O., Tunyasuvunakool, K., Bates, R., Žídek, A., Potapenko, A., Bridgland, A., Meyer, C., Kohl, S. A. A., Ballard, A. J., Cowie, A., Romera-Paredes, B., Nikolov, S., Jain, R., Adler, J., Back, T., Petersen, S., Reiman, D., Clancy, E., Zielinski, M., Steinegger, M., Pacholska, M., Berghammer, T., Bodenstein, S., Silver, D., Vinyals, O., Senior, A. W., Kavukcuoglu, K., Kohli, P. & Hassabis, D. (2021). *Nature*, **596**, 583–589. 10.1038/s41586-021-03819-2 PMC8371605 34265844

[bb40] E, J., Kim, Y., Bielecki, J., Sikorski, M., de Wijn, R., Fortmann-Grote, C., Sztuk-Dambietz, J., Koliyadu, J. C. P., Letrun, R., Kirkwood, H. J., Sato, T., Bean, R., Mancuso, A. P. & Kim, C. (2022). *Struct. Dyn.* **9**, 064101. 10.1063/4.0000169 PMC9675053 36411869

[bb41] Kurta, R. P., Donatelli, J. J., Yoon, C. H., Berntsen, P., Bielecki, J., Daurer, B. J., DeMirci, H., Fromme, P., Hantke, M. F., Maia, F. R. N. C., Munke, A., Nettelblad, C., Pande, K., Reddy, H. K. N., Sellberg, J. A., Sierra, R. G., Svenda, M., van der Schot, G., Vartanyants, I. A., Williams, G. J., Xavier, P. L., Aquila, A., Zwart, P. H. & Mancuso, A. P. (2017). *Phys. Rev. Lett.* **119**, 158102. 10.1103/PhysRevLett.119.158102 PMC5757528 29077445

[bb42] Latychevskaia, T. (2018). *Appl. Opt.* **57**, 7187–7197. 10.1364/AO.57.007187 30182978

[bb43] Loh, N. D. & Elser, V. (2009). *Phys. Rev. E*, **80**, 026705. 10.1103/PhysRevE.80.026705 19792279

[bb44] Lundholm, I. V., Sellberg, J. A., Ekeberg, T., Hantke, M. F., Okamoto, K., van der Schot, G., Andreasson, J., Barty, A., Bielecki, J., Bruza, P., Bucher, M., Carron, S., Daurer, B. J., Ferguson, K., Hasse, D., Krzywinski, J., Larsson, D. S. D., Morgan, A., Mühlig, K., Müller, M., Nettelblad, C., Pietrini, A., Reddy, H. K. N., Rupp, D., Sauppe, M., Seibert, M., Svenda, M., Swiggers, M., Timneanu, N., Ulmer, A., Westphal, D., Williams, G., Zani, A., Faigel, G., Chapman, H. N., Möller, T., Bostedt, C., Hajdu, J., Gorkhover, T. & Maia, F. R. N. C. (2018). *IUCrJ*, **5**, 531–541. 10.1107/S2052252518010047 PMC6126651 30224956

[bb45] Lunin, V. Y., Lunina, N. L., Petrova, T. E., Skovoroda, T. P., Urzhumtsev, A. G. & Podjarny, A. D. (2000). *Acta Cryst.* D**56**, 1223–1232. 10.1107/s0907444900010088 10998618

[bb46] Marchesini, S., He, H., Chapman, H. N., Hau-Riege, S. P., Noy, A., Howells, M. R., Weierstall, U. & Spence, J. C. H. (2003). *Phys. Rev. B*, **68**, 140101.

[bb47] Miao, J., Hodgson, K. O. & Sayre, D. (2001). *Proc. Natl Acad. Sci. USA*, **98**, 6641–6645. 10.1073/pnas.111083998 PMC34406 11390993

[bb48] Miao, J., Nishino, Y., Kohmura, Y., Johnson, B., Song, C., Risbud, S. H. & Ishikawa, T. (2005). *Phys. Rev. Lett.* **95**, 085503. 10.1103/PhysRevLett.95.085503 16196870

[bb49] Miao, J. W., Charalambous, P., Kirz, J. & Sayre, D. (1999). *Nature*, **400**, 342–344.

[bb50] Millán, C., Keegan, R. M., Pereira, J., Sammito, M. D., Simpkin, A. J., McCoy, A. J., Lupas, A. N., Hartmann, M. D., Rigden, D. J. & Read, R. J. (2021). *Proteins*, **89**, 1752–1769. 10.1002/prot.26214 PMC8881082 34387010

[bb51] Mosalaganti, S., Obarska-Kosinska, A., Siggel, M., Taniguchi, R., Turoňová, B., Zimmerli, C. E., Buczak, K., Schmidt, F. H., Margiotta, E., Mackmull, M., Hagen, W. J. H., Hummer, G., Kosinski, J. & Beck, M. (2022). *Science*, **376**, eabm9506. 10.1126/science.abm9506 35679397

[bb52] Munke, A., Andreasson, J., Aquila, A., Awel, S., Ayyer, K., Barty, A., Bean, R. J., Berntsen, P., Bielecki, J., Boutet, S., Bucher, M., Chapman, H. N., Daurer, B. J., DeMirci, H., Elser, V., Fromme, P., Hajdu, J., Hantke, M. F., Higashiura, A., Hogue, B. G., Hosseinizadeh, A., Kim, Y., Kirian, R. A., Reddy, H. K. N., Lan, T. Y., Larsson, D. S. D., Liu, H. G., Loh, N. D., Maia, F. R. N. C., Mancuso, A. P., Mühlig, K., Nakagawa, A., Nam, D., Nelson, G., Nettelblad, C., Okamoto, K., Ourmazd, A., Rose, M., van der Schot, G., Schwander, P., Seibert, M. M., Sellberg, J. A., Sierra, R. G., Song, C. Y., Svenda, M., Timneanu, N., Vartanyants, I. A., Westphal, D., Wiedorn, M. O., Williams, G. J., Xavier, P. L., Yoon, C. H. & Zook, J. (2016). *Sci. Data*, **3**, 160064.

[bb53] Neutze, R., Wouts, R., van der Spoel, D., Weckert, E. & Hajdu, J. (2000). *Nature*, **406**, 752–757. 10.1038/35021099 10963603

[bb54] Nishino, Y., Miao, J. & Ishikawa, T. (2003). *Phys. Rev. B*, **68**, 220101.

[bb55] Penczek, P. A., Grassucci, R. A. & Frank, J. (1994). *Ultramicroscopy*, **53**, 251–270. 10.1016/0304-3991(94)90038-8 8160308

[bb56] Pettersen, E. F., Goddard, T. D., Huang, C. C., Couch, G. S., Greenblatt, D. M., Meng, E. C. & Ferrin, T. E. (2004). *J. Comput. Chem.* **25**, 1605–1612. 10.1002/jcc.20084 15264254

[bb57] Poudyal, I., Schmidt, M. & Schwander, P. (2020). *Struct. Dyn.* **7**, 024102. 10.1063/1.5144516 PMC7088463 32232074

[bb58] Powell, H. R. (1999). *Acta Cryst.* D**55**, 1690–1695. 10.1107/s0907444999009506 10531518

[bb59] Punjani, A., Rubinstein, J. L., Fleet, D. J. & Brubaker, M. A. (2017). *Nat. Methods*, **14**, 290–296. 10.1038/nmeth.4169 28165473

[bb60] Reddy, H. K. N., Yoon, C. H., Aquila, A., Awel, S., Ayyer, K., Barty, A., Berntsen, P., Bielecki, J., Bobkov, S., Bucher, M., Carini, G. A., Carron, S., Chapman, H., Daurer, B., DeMirci, H., Ekeberg, T., Fromme, P., Hajdu, J., Hanke, M. F., Hart, P., Hogue, B. G., Hosseinizadeh, A., Kim, Y., Kirian, R. A., Kurta, R. P., Larsson, D. S. D., Loh, N. D., Maia, F. R. N. C., Mancuso, A. P., Muhlig, K., Munke, A., Nam, D., Nettelblad, C., Ourmazd, A., Rose, M., Schwander, P., Seibert, M., Sellberg, J. A., Song, C., Spence, J. C. H., Svenda, M., Van der Schot, G., Vartanyants, I. A., Williams, G. J. & Xavier, P. L. (2017). *Sci. Data*, **4**, 170079. 10.1038/sdata.2017.79 PMC5501160 28654088

[bb61] Rosenthal, P. B. & Henderson, R. (2003). *J. Mol. Biol.* **333**, 721–745. 10.1016/j.jmb.2003.07.013 14568533

[bb62] Scheres, S. H. W. (2012). *J. Struct. Biol.* **180**, 519–530. 10.1016/j.jsb.2012.09.006 PMC3690530 23000701

[bb63] Seibert, M. M., Ekeberg, T., Maia, F. R. N. C., Svenda, M., Andreasson, J., Jönsson, O., Odić, D., Iwan, B., Rocker, A., Westphal, D., Hantke, M., DePonte, D. P., Barty, A., Schulz, J., Gumprecht, L., Coppola, N., Aquila, A., Liang, M. N., White, T. A., Martin, A., Caleman, C., Stern, S., Abergel, C., Seltzer, V., Claverie, J. M., Bostedt, C., Bozek, J. D., Boutet, S., Miahnahri, A. A., Messerschmidt, M., Krzywinski, J., Williams, G., Hodgson, K. O., Bogan, M. J., Hampton, C. Y., Sierra, R. G., Starodub, D., Andersson, I., Bajt, S., Barthelmess, M., Spence, J. C. H., Fromme, P., Weierstall, U., Kirian, R., Hunter, M., Doak, R. B., Marchesini, S., Hau-Riege, S. P., Frank, M., Shoeman, R. L., Lomb, L., Epp, S. W., Hartmann, R., Rolles, D., Rudenko, A., Schmidt, C., Foucar, L., Kimmel, N., Holl, P., Rudek, B., Erk, B., Hömke, A., Reich, C., Pietschner, D., Weidenspointner, G., Strüder, L., Hauser, G., Gorke, H., Ullrich, J., Schlichting, I., Herrmann, S., Schaller, G., Schopper, F., Soltau, H., Kühnel, K. U., Andritschke, R., Schröter, C. D., Krasniqi, F., Bott, M., Schorb, S., Rupp, D., Adolph, M., Gorkhover, T., Hirsemann, H., Potdevin, G., Graafsma, H., Nilsson, B., Chapman, H. N. & Hajdu, J. (2011). *Nature*, **470**, 78–81.

[bb64] Shapiro, D., Thibault, P., Beetz, T., Elser, V., Howells, M., Jacobsen, C., Kirz, J., Lima, E., Miao, H., Neiman, A. M. & Sayre, D. (2005). *Proc. Natl Acad. Sci. USA*, **102**, 15343–15346. 10.1073/pnas.0503305102 PMC1250270 16219701

[bb65] Shneerson, V. L., Ourmazd, A. & Saldin, D. K. (2008). *Acta Cryst.* A**64**, 303–315. 10.1107/S0108767307067621 18285625

[bb66] Skalidis, I., Kyrilis, F. L., Tüting, C., Hamdi, F., Chojnowski, G. & Kastritis, P. L. (2022). *Structure*, **30**, 575–589.e6. 10.1016/j.str.2022.01.001 35093201

[bb67] Sobolev, E., Zolotarev, S., Giewekemeyer, K., Bielecki, J., Okamoto, K., Reddy, H. K. N., Andreasson, J., Ayyer, K., Barak, I., Bari, S., Barty, A., Bean, R., Bobkov, S., Chapman, H. N., Chojnowski, G., Daurer, B. J., Dörner, K., Ekeberg, T., Flückiger, L., Galzitskaya, O., Gelisio, L., Hauf, S., Hogue, B. G., Horke, D. A., Hosseinizadeh, A., Ilyin, V., Jung, C., Kim, C., Kim, Y., Kirian, R. A., Kirkwood, H., Kulyk, O., Küpper, J., Letrun, R., Loh, N. D., Lorenzen, K., Messerschmidt, M., Mühlig, K., Ourmazd, A., Raab, N., Rode, A. V., Rose, M., Round, A., Sato, T., Schubert, R., Schwander, P., Sellberg, J. A., Sikorski, M., Silenzi, A., Song, C. Y., Spence, J. C. H., Stern, S., Sztuk-Dambietz, J., Teslyuk, A., Timneanu, N., Trebbin, M., Uetrecht, C., Weinhausen, B., Williams, G. J., Xavier, P. L., Xu, C., Vartanyants, I. A., Lamzin, V. S., Mancuso, A. & Maia, F. R. N. C. (2020). *Commun. Phys.* **3**, 97.

[bb68] Subbiah, S. (1991). *Science*, **252**, 128–133. 10.1126/science.2011749 2011749

[bb69] Tegze, M. & Bortel, G. (2012). *J. Struct. Biol.* **179**, 41–45. 10.1016/j.jsb.2012.04.014 22575364

[bb70] Tegze, M. & Bortel, G. (2021). *IUCrJ*, **8**, 980–991. 10.1107/S205225252100868X PMC8562656 34804550

[bb71] Tejero, R., Huang, Y. J., Ramelot, T. A. & Montelione, G. T. (2022). *Front. Mol. Biosci.* **9**, 877000. 10.3389/fmolb.2022.877000 PMC9234698 35769913

[bb72] Tunyasuvunakool, K., Adler, J., Wu, Z., Green, T., Zielinski, M., Žídek, A., Bridgland, A., Cowie, A., Meyer, C., Laydon, A., Velankar, S., Kleywegt, G. J., Bateman, A., Evans, R., Pritzel, A., Figurnov, M., Ronneberger, O., Bates, R., Kohl, S. A. A., Potapenko, A., Ballard, A. J., Romera-Paredes, B., Nikolov, S., Jain, R., Clancy, E., Reiman, D., Petersen, S., Senior, A. W., Kavukcuoglu, K., Birney, E., Kohli, P., Jumper, J. & Hassabis, D. (2021). *Nature*, **596**, 590–596. 10.1038/s41586-021-03828-1 PMC8387240 34293799

[bb73] Yefanov, O. M. & Vartanyants, I. A. (2013). *J. Phys. B At. Mol. Opt. Phys.* **46**, 164013.

[bb74] Zhao, H., Zhang, H., She, Z., Gao, Z., Wang, Q., Geng, Z. & Dong, Y. (2023). *Int. J. Mol. Sci.* **24**, 2740. 10.3390/ijms24032740 PMC9916901 36769074

