# Supplementary material for: A predicted model-aided reconstruction algorithm for X-ray free-electron laser single-particle imaging
Source: IUCrJ. 2024 Jun 21;11(Pt 4):602–19. doi: 10.1107/S2052252524004858 (PMC11220885; doi:10.1107/S2052252524004858)
Supplement: Supplementary file 3 [file m-11-00602-sup3.pdf]

IUCrJ

Volume 11 (2024)

Supporting information for article:

A predicted model-aided reconstruction algorithm for X-ray free-electron laser single-particle imaging

Zhichao Jiao, Yao He, Xingke Fu, Xin Zhang, Zhi Geng and Wei Ding

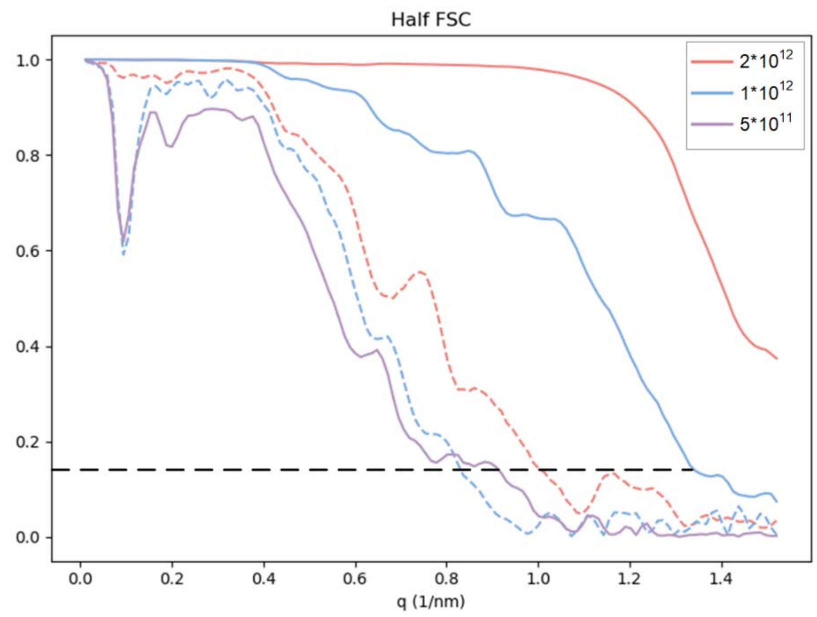

Figure S1 A comparison of half FSC of the recovered electron density maps using prior phases (solid lines) and random phases (dotted lines) under different pulse fluences, plotted against the length of scattering vector. Tests were conducted using the protein 6ZFP. The horizontal line indicates the resolution of the recovered 3D electron density map, where FSC drops to 0.14. Phase recovery using half data set is failed when the pulse photon number is less than $1*10^{12}$ in random phases and less than $5*10^{11}$ in prior phases.

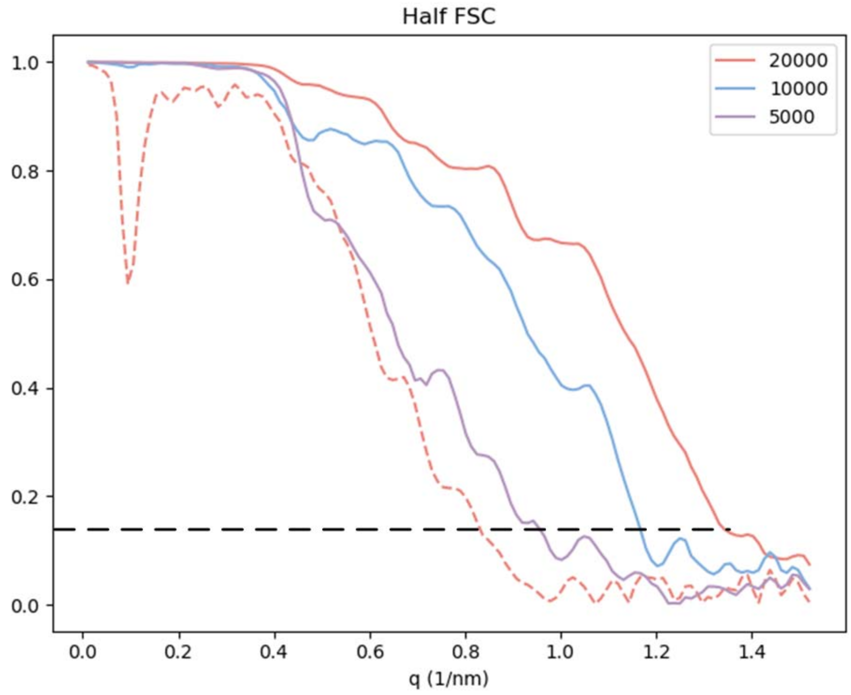

Figure S2 A comparison of half FSC of the recovered electron density maps using prior phases (solid lines) and random phases (dotted lines) under different pattern numbers, plotted against the length of scattering vector. Tests were conducted using the protein 6ZFP. The horizontal line indicates the resolution of the recovered 3D electron density map, where FSC drops to 0.14. Phase recovery using half data set is failed when the pattern number is less than 20,000 in random phases and less than 5,000 in prior phases.

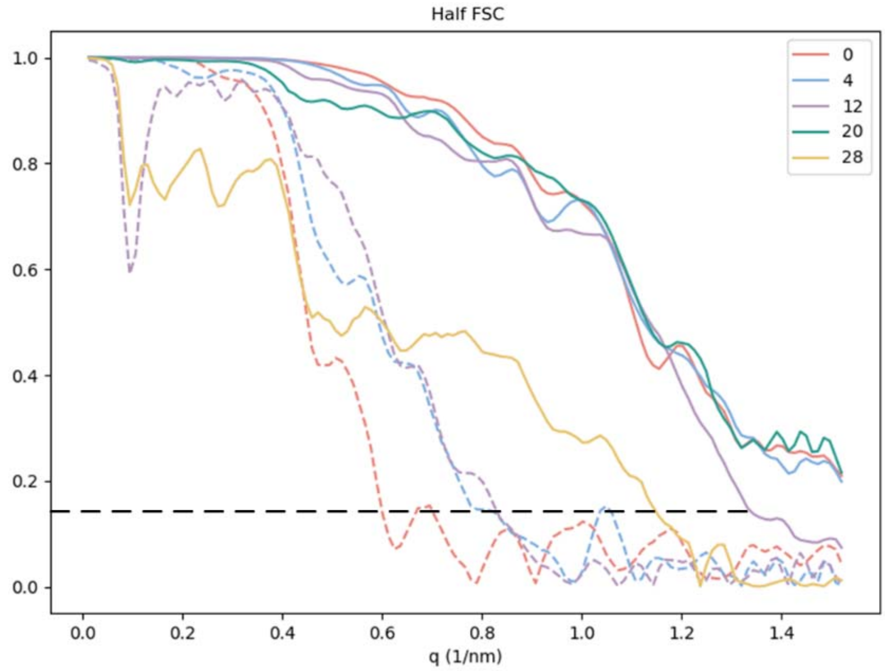

Figure S3 A comparison of half FSC of the recovered electron density maps using prior phases (solid lines) and random phases (dotted lines) under different beam stop size, plotted against the length of scattering vector. Tests were conducted using the protein 6ZFP. The horizontal line indicates the resolution of the recovered 3D electron density map, where FSC drops to 0.14. Phase recovery using half data set is failed when the beam stop size is higher than 12*12 pix in random phases and higher than 28*28 pix in prior phases.

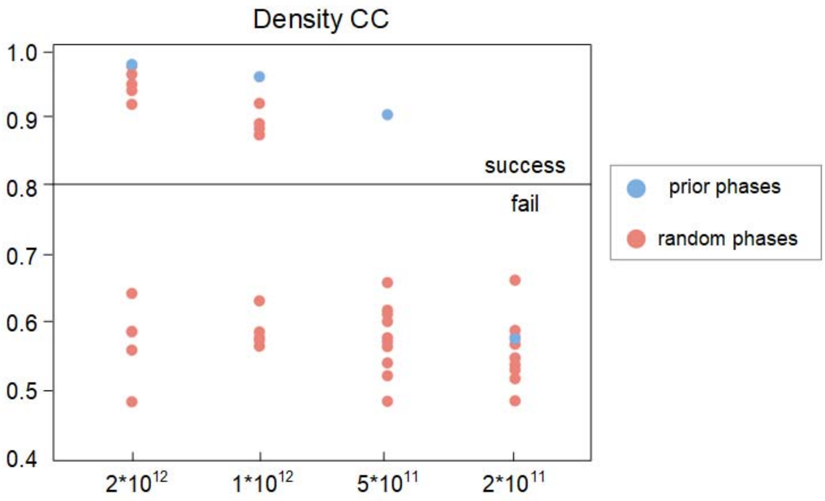

Figure S4 The overall correlation coefficient between the real density map and the recovered density map under different pulse photon numbers. Tests were conducted using the protein 6ZFP. Blue dots represent prior phases, while magenta dots indicate random phases. A threshold of 0.8 is used to determine the successful reconstruction of electron densities.

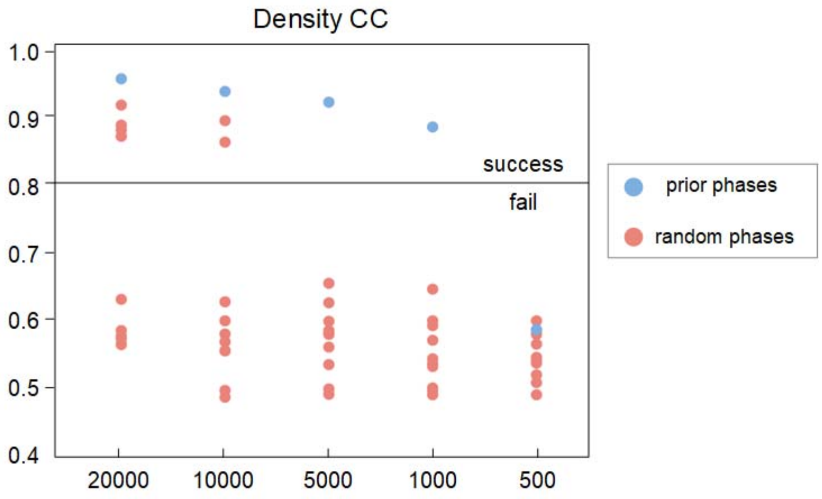

Figure S5 The overall correlation coefficient between the real density map and the recovered density map using different number of patterns. Tests were conducted using the protein 6ZFP. Blue dots represent prior phases, while magenta dots indicate random phases. A threshold of 0.8 is used to determine the successful reconstruction of electron densities.

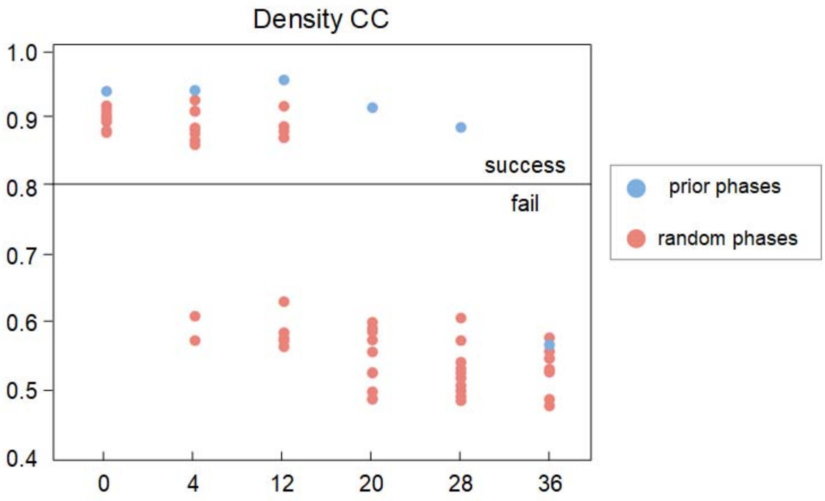

Figure S6 The overall correlation coefficient between the real density map and the recovered density map in different beam stop sizes. Tests were conducted using the protein 6ZFP. Blue dots represent prior phases, while magenta dots indicate random phases. A threshold of 0.8 is used to determine the successful reconstruction of electron densities.

Table S1 Success rates of iterative phasing using prior phases and random phases under different pulse fluences

Number of photons per pulse	Beam focus size (μm)	Prior phases (1100ER)	Random phases (1000HIO + 100ER)	Prior phases (1000HIO + 100ER)	Random phases (1100ER)
$2*10^{12}$	0.1	1/1	5/10	1/1	0/10
$1*10^{12}$	0.1	1/1	5/10	1/1	0/10
$5*10^{11}$	0.1	1/1	0/10	0/1	0/10
$2*10^{11}$	0.1	0/1	0/10	0/1	0/10

Table S2 Success rates of iterative phasing using prior phases and random phases using different number of patterns

No. of patterns	Prior phases (1100ER)	Random phases (1000HIO + 100ER)	Prior phases (1000HIO + 100ER)	Random phases (1100ER)
20000	1/1	5/10	1/1	0/10
10000	1/1	2/10	0/1	0/10
5000	1/1	0/10	0/1	0/10
1000	1/1	0/10	0/1	0/10
500	0/1	0/10	0/1	0/10

Table S3 Success rates of iterative phasing using prior phases and random phases under different sizes of beam stop

Beam stop (pixs)	Prior phases (1100ER)	Random phases (1000HIO + 100ER)	Prior phases (1000HIO + 100ER）	Random phases (1100ER）
0	1/1	10/10	1/1	0/10
4	1/1	8/10	1/1	0/10
12	1/1	5/10	0/1	0/10
20	1/1	0/10	0/1	0/10
28	1/1	0/10	0/1	0/10
36	0/1	0/10	0/1	0/10
